# Structural basis of α_1A_-adrenergic receptor activation and recognition by an extracellular nanobody

**DOI:** 10.1038/s41467-023-39310-x

**Published:** 2023-06-20

**Authors:** Yosuke Toyoda, Angqi Zhu, Fang Kong, Sisi Shan, Jiawei Zhao, Nan Wang, Xiaoou Sun, Linqi Zhang, Chuangye Yan, Brian K. Kobilka, Xiangyu Liu

**Affiliations:** 1 School of Medicine, Tsinghua University, Beijing, 100084 China; 2 Beijing Frontier Research Center for Biological Structure, Beijing Advanced Innovation Center for Structural Biology, Tsinghua University, Beijing, 100084 China; 3 State Key Laboratory of Membrane Biology, Tsinghua-Peking Center for Life Sciences, School of Life Sciences, Tsinghua University, Beijing, 100084 China; 4 NexVac Research Center, Comprehensive AIDS Research Center, Center for Infectious Disease Research, Tsinghua University, Beijing, 100084 China; 5 Department of Molecular and Cellular Physiology, Stanford University School of Medicine, Stanford, CA 94305 USA; 6 State Key Laboratory of Membrane Biology, Tsinghua-Peking Center for Life Sciences, School of Pharmaceutical Sciences, Tsinghua University, Beijing, 100084 China; 7 Present Address: Institute for Integrated Cell-Material Sciences, Institute for Advanced Study, Kyoto University, Kyoto, 606-8501 Japan

**Keywords:** Cryoelectron microscopy, Receptor pharmacology, G protein-coupled receptors

## Abstract

The α_1A-_adrenergic receptor (α_1A_AR) belongs to the family of G protein-coupled receptors that respond to adrenaline and noradrenaline. α_1A_AR is involved in smooth muscle contraction and cognitive function. Here, we present three cryo-electron microscopy structures of human α_1A_AR bound to the endogenous agonist noradrenaline, its selective agonist oxymetazoline, and the antagonist tamsulosin, with resolutions range from 2.9 Å to 3.5 Å. Our active and inactive α_1A_AR structures reveal the activation mechanism and distinct ligand binding modes for noradrenaline compared with other adrenergic receptor subtypes. In addition, we identified a nanobody that preferentially binds to the extracellular vestibule of α_1A_AR when bound to the selective agonist oxymetazoline. These results should facilitate the design of more selective therapeutic drugs targeting both orthosteric and allosteric sites in this receptor family.

## Abstract (web summary)

α_1A_-adrenergic receptor (α_1A_AR) regulates smooth muscle contraction and cognitive functions. Here, authors provide structural insight into α_1A_AR activation and binding modes of the orthosteric ligands and an extracellular allosteric nanobody.

## Introduction

Adrenergic receptors (ARs), which mediate physiological responses to the neurotransmitter noradrenaline (norepinephrine) and the hormone adrenaline (epinephrine), are family A G protein-coupled receptors (GPCRs). They mediate responses to sympathetic nervous system activation and are subdivided into α_1_ (α_1A_, α_1B_ and α_1D_), α_2_ (α_2A_, α_2B_ and α_2c_) and β (β_1_, β_2,_ and β_3_) ARs. α_1_ARs are predominantly coupled to the heterotrimeric G_q/11_ family of G proteins, leading to the activation of phospholipase C and the increase of cytosolic Ca^2+^. α_1A_AR was cloned from the bovine brain and initially designated α_1C_AR^1^. As the original α_1A_AR and α_1D_AR appeared to represent the same subtypes, these clones have been renamed α_1A_AR (formerly α_1C_AR), α_1B_AR (formerly α_1B_AR) and α_1D_AR (formerly α_1A_AR and α_1D_AR)^2,3^. α_1_ARs are expressed in a wide range of tissues including blood vessels, kidney, spleen, liver, brain and lower urinary tract^2,3^. In the periphery, postsynaptic α_1_AR activation mediates smooth muscle contraction, therefore the selective α_1A_AR agonist oxymetazoline^4^ is clinically used for the treatment of nasal congestion, whereas selective α_1A_AR antagonists such as tamsulosin and silodosin are prescribed to treat hypertension and benign prostatic hyperplasia^5^ (Supplementary Fig. 1). As α_1_AR plays a role in regulating synaptic plasticity and memory consolidation^2^, the α_1_AR antagonist prazosin is used to reduce nightmares and overall Post Traumatic Stress Disorder symptoms^6^ and has a potential for the preventing cytokine storm syndrome caused by the severe acute respiratory syndrome coronavirus 2, a leading cause of morbidity and mortality in coronavirus disease 2019^7^.

Recent progress in the structural characterization of GPCRs including the adrenergic receptors has clarified the mechanism of ligand recognition and G protein activation. The βARs are extensively well-characterized GPCRs and a number of structures have been determined in the active and inactive states^8–13^. Moreover, recent structures of active and inactive α_2A_AR^14,15^, active α_2B_AR-G_i/o_^16^ and inactive α_2C_AR^17^ demonstrated the subtype selectivity of ligand recognition between α_2_ARs and βARs. However, little is known about the structure and mechanism of activation of the α_1_AR subtypes. The only available structure is the inactive α_1B_AR structure^18^. Here we present three cryo-electron microscopy (cryo-EM) structures of active α_1A_AR bound to oxymetazoline and the endogenous agonist noradrenaline, along with inactive α_1A_AR bound to tamsulosin. We also discovered a nanobody (a single domain antibody) Nb29 that binds to the extracellular vestibule of the agonist-binding pocket. Antibodies against GPCRs have attracted particular interest for pharmaceutical applications^19^, and are useful research tools for stabilizing GPCR conformations for structural analysis^20,21^. Nevertheless, only a few class A GPCR structures in complex with the extracellular antibody are available^22–26^. These findings may guide the development of more effective drugs for the α_1A_AR.

## Results

### Structure determination of active and inactive α_1A_AR

**Fig. 1:**
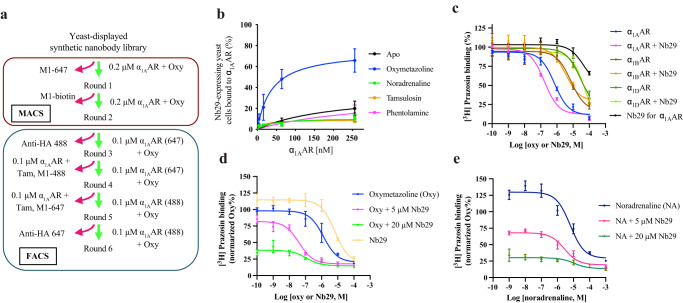
Selection and characterization of Nb29 as a conformationally selective nanobody for α_1A_AR. **a** Flow chart of the selection process of conformationally selective nanobodies from the yeast-displayed nanobody library. For rounds 1 and 2, 0.2 μM α_1A_AR bound to oxymetazoline (oxy) was used for selection and alexa647-labeled anti-FLAG M1 antibody (M1-647) or biotin-labeled anti-FLAG M1 antibody Fab fragment (M1-biotin) was used for the preclear. For the FACS selection, different combinations of counterselection were performed using oxymetazoline, tamsulosin (Tam), M1-488/647, and anti-HA antibodies. **b** On-yeast titration to estimate the affinity of Nb29 for α_1A_AR, evaluated by flow cytometry. The ratio of Nb29-displayed yeast cells bound purified α_1A_AR in the presence or absence of 500 μM ligands was analyzed. The data represent mean ± s.e.m. of *n* = 3 independent measurements. **c** ^3^H-prazosin radioligand competition binding of α_1_AR subtype for oxymetazoline in *Sf9* membranes. Samples in the presence of Nb29 were used at 5 μM concentration of Nb29. The data represent mean ± s.e.m. of *n* = 3 independent measurements. **d, e** ^3^H-prazosin radioligand competition binding of the purified α_1A_AR-bound M1-Flag affinity resin for oxymetazoline (Oxy), noradrenaline (NA) or Nb29. The data represent mean ± s.e.m. of *n* = 3 (NA + 5 μM Nb29), and *n* = 6 (the others) independent measurements. Binding affinity values are provided in Supplementary Table 1. Source data are provided in the Source Data file.

We expressed human α_1A_AR in baculovirus-infected *Spodoptera frugiperda* (*Sf9*) insect cells. We constructed a variant of human α_1A_AR lacking residues 371–466 in the C-terminus, and three *N*-linked glycosylation sites (N7Q, N13Q and N22Q) in the N-terminus were mutated. To further stabilize the receptor, we discovered a conformationally selective nanobody from a library of synthetic nanobodies displayed on the surface of *Saccharomyces cerevisiae*^27^. After two rounds of magnetic-activated cell sorting (MACS) and four rounds of fluorescence-activated cell sorting (FACS) with oxymetazoline- and tamsulosin-bound α_1A_AR, we identified Nb29 as the most enriched clone (Fig. 1a and Supplementary Fig. 2; See Method). An on-yeast titration assay indicated that Nb29 has selectivity for oxymetazoline-bound α_1A_AR compared with apo, noradrenaline-, and antagonist (tamsulosin and phentolamine)-bound states (Fig. 1b and Supplementary Table 1a). In a ligand binding assay, Nb29 induced the left shift of the agonist competition curves for α_1A_AR over α_1B_- and α_1D_AR (Fig. 1c and Supplementary Table 1b). Although Nb29 on its own competed for the antagonist [^3^H]prazosin binding for α_1A_AR and might affect the competition binding results, we observed a larger left shift of oxymetazoline competition curves than those for noradrenaline, which is in agreement with the on-yeast titration result (Fig. 1d, e and Supplementary Table 1c).

**Fig. 2:**
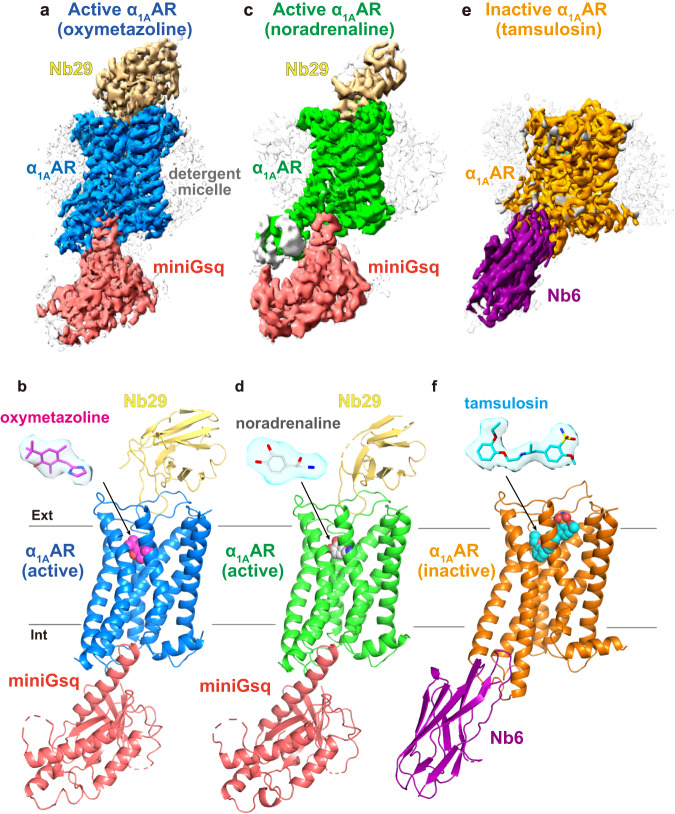
Cryo-EM structures of oxymetazoline- and noradrenaline-bound Nb29–α_1A_AR-miniGsq and tamsulosin-bound α_1A_AR-Nb6 complexes. The cryo-EM density maps and structure models of the Nb29-α_1A_AR-miniGsq complexes bound to the agonists oxymetazoline (**a**, **b**) and noradrenaline (**c**, **d**), and α_1A_AR-Nb6 complex bound to the antagonist tamsulosin (**e**, **f**). The detergent micelle (**a**, **c**, **e**) and unmodelled mT4L (**b**) are shown in gray. The densities of the ligands (shown as sticks) are depicted as surfaces. Color code for the proteins is as follows: oxymetazoline-bound active α_1A_AR (blue), noradrenaline-bound active α_1A_AR (green), inactive α_1A_AR (orange), miniGsq (pink), Nb29 (yellow), and Nb6 (purple). Small molecules are colored as follows: oxymetazoline in magenta, noradrenaline in gray, and tamsulosin in cyan.

To solve the structure, we formed the α_1A_AR complex with Nb29 and obtained the initial cryo-EM structure at 4 Å resolution, and found that Nb29 binds to the extracellular region of the α_1A_AR. To further stabilize the receptor, we used α_1A_AR construct fused with a minimal-T4 lysozyme (mT4L) in the intracellular loop 3 (ICL3)^28^ (Supplementary Fig. 3a), and formed a complex with engineered minimal Gsq protein (miniGsq) in which mini-Gs was substituted with the 15 residues of carboxyl-terminal α5 helix of Gq protein^29^ (Methods and Supplementary Fig. 3b, c). The α_1A_AR in complex with the heterotrimeric G_q/11_ proteins was not stable enough for structure determination. Finally, we obtained the cryo-EM structures of active α_1A_AR bound with oxymetazoline (Nb29-α_1A_AR-miniGsq) at a global resolution of 2.9 Å (Fig. 2a, b; Supplementary Figs. 3d–f and 4a; Supplementary Table 2). The cryo-EM map allowed the model building of most of the regions with a clear electron density for the ligand. The map density of the extracellular region of α_1A_AR is relatively clear because of the bound Nb29, whereas there is poor density for the fused mT4L due to map refinement by masking out the mT4L. Subsequently, we also solved the Nb29-α_1A_AR-miniGsq complex bound with noradrenaline at a global resolution of 3.5 Å (Fig. 2c, d; Supplementary Figs. 3g–i and 4b; Supplementary Table 2). Although the map quality allowed the model building of the receptor with clear electron density for the ligand, the map density for Nb29 is weak. The weaker map density is in agreement with the observation that Nb29 preferentially binds to oxymetazoline-bound receptors over noradrenaline-bound receptors (Fig. 1b). The resolution of the Nb29-dissociated α_1A_AR-miniGsq complex is much lower (~6 Å) than that of the Nb29-bound complex (Supplementary Fig. 3h).

To solve the inactive α_1A_AR structure, our crystallographic and cryo-EM experiments using fusion protein and/or the other nanobodies selected from the synthetic nanobody library were unsuccessful. Thus, we utilized a recently described engineering strategy to enable the binding of nanobody 6 (Nb6) that engages the ICL3 of the inactive-state κ-opioid receptor (kOR)^21^. Based on the cryo-EM structure of engineered neurotensin receptor 1 (NTSR1)-Nb6 complex, we swapped the same site from C205^5.59^ of transmembrane (TM) 5 (T247^5.59^ of kOR) to G275^6.38^ of TM6 (L277^6.38^ of kOR) including intracellular loop (ICL) 3 (superscripts indicate Ballesteros-Weinstein numbering for GPCRs^30^). In addition, we introduced two thermostabilizing point mutations, S113R^3.39^ to mimic allosteric sodium ion binding^31^, and M115W^3.41^ to increase expression^32^. These mutations were used for solving other inactive GPCR structures such as prostaglandin E receptor EP4 for the R^3.39^ mutation^22^, and dopamine D2 receptor for double mutation of positions 3.39 and 3.41^25^. In radioligand binding studies, the α_1A_AR-kOR mutant showed enhancement of [^3^H]prazosin binding by Nb6, but did not significantly alter the binding affinities for the agonist oxymetazoline or the antagonist tamsulosin (Supplementary Fig. 5a, b). We formed the α_1A_AR-Nb6 complex and solved the cryo-EM structure of the inactive α_1A_AR bound to tamsulosin at a global resolution of 3.3 Å resolution. (Fig. 2e, f; Supplementary Fig. 5c–h; Supplementary Table 2). The cryo-EM map allowed the model building of most of the regions with a clear electron density for tamsulosin; the map density of the inactive α_1A_AR-Nb6 complex is relatively weak for ECL2 and well-defined in the cytoplasmic region including Nb6 binding domain where ICL3 and the cytoplasmic sides of TM5 and 6 were exchanged to those of the kOR. Although Nb6 binds to a similar site as in the kOR-Nb6 complex^33^ and locks the conformation of the TM5 and TM6 of α_1A_AR, we observed fewer polar interactions in our structure when compared with the kOR-Nb6 and NTSR1-Nb6 complexes^21^ (Supplementary Fig. 6a–e). The active α_1A_AR structures enable us to model a putative cholesteryl hemisuccinate (CHS) molecule bordering TM3-5 in the active structures, in contrast, we observed only a weak density in the inactive α_1A_AR structure, since the side chain of M115W^3.41^ overlaps the regions corresponding to the lipid tail of CHS (Supplementary Fig. 6g, h). The side chain of S113R^3.39^ is located in the putative sodium ion binding site as designed (Supplementary Fig. 6i, j)^22,31^.

**Fig. 3:**
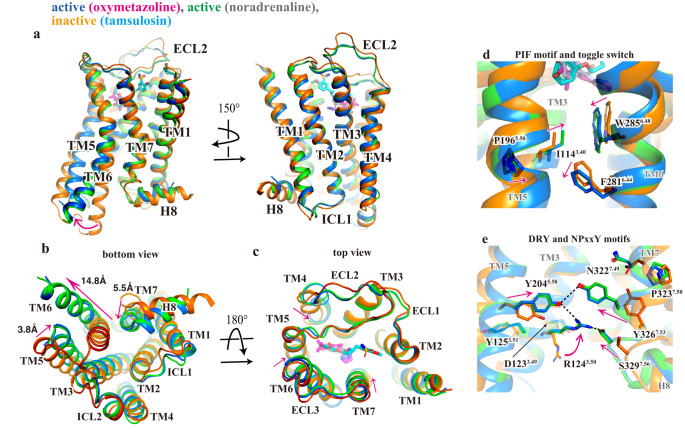
Structural comparison between active and inactive α_1A_AR. **a** Comparison of α_1A_AR between oxymetazoline-bound (blue), noradrenaline-bound (green), and inactive (orange) states viewed from the side. **b** Intracellular view of superposed α_1A_ARs. Distances were measured between the Cα atoms of E269^6.30^ (E269L^6.30^ in α_1A_AR-kOR) in TM6, K212^5.66^ in TM5, and C328^7.55^ in TM7. **c** Extracellular view of superposed α_1A_ARs. The maximum distances of the side chain displacement of W285 (position 7th carbon of the indole ring) are 2.4 Å between the noradrenaline-bound active state and the tamsulosin-bound inactive state, and 1.8 Å between the oxymetazoline-bound state and the tamsulosin-bound state. **d** Conformational change of PIF motif and toggle switch W285^6.48^. **e** Conformational change of DRY and NpxxY motifs. Conformational changes upon activation are shown with magenta arrows. Hydrogen bonds are shown as black dashed lines.

Structural comparison of active and inactive states of α_1A_AR exhibits 14.5 Å outward displacement of an intracellular segment of TM6 that is characteristic of receptor activation (Fig. 3a–c). The TM6 movement is accompanied by a small rotation of the helix, as well as inward movements of TMs 3, 5, and 7 toward TM6. α_1A_AR also exhibits other characteristics of the activation of class A GPCRs^9,14,16^. We observed the displacement of the side chain of W285^6.48^ (Fig. 3d), a highly conserved residue that contributes to conformational changes associated with activation for some GPCRs. We also observe conformational changes in the conserved PIF (P196^5.50^ I114^3.40^ and F260^6.44^) interaction, as well as the NPxxY (N322^7.49^, P323^7.50^, and Y326^7.53^) and DRY (D123^3.49^, R124^3.50^ and Y125^3.51^) motifs (Fig. 3d, e). In the active α_1A_AR, R124^3.50^ forms hydrogen bond networks with Y204^5.58^, Y326^7.53^ and C329^7.56^ (Fig. 3e). These structural changes allow the C-terminal helix (α5 helix) of Gα to engage the receptor core, as described below. In the extracellular view, due to the Nb29 binding, the conformation of ECL2, TMs 4 and 7 in the active state is closer to the receptor core, as discussed later in detail.

### Orthosteric ligand-binding pocket of α_1A_AR

**Fig. 4:**
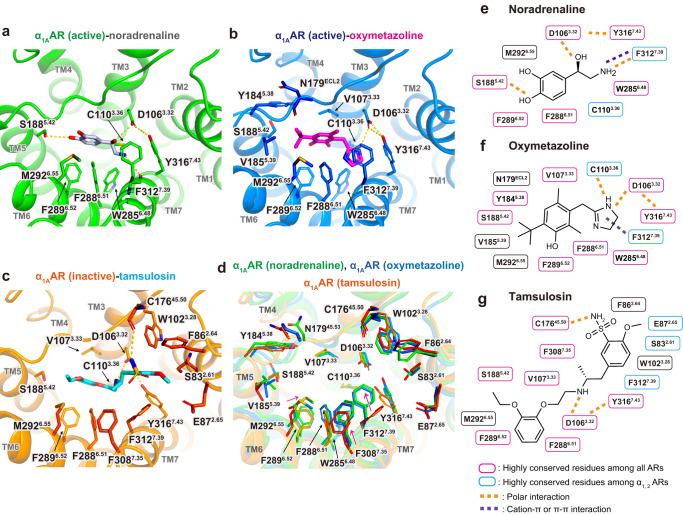
Orthosteric ligand-binding pocket of α_1A_AR. Side view of α_1A_AR ligand-binding site of noradrenaline (**a**), oxymetazoline (**b**), and tamsulosin (**c**). Residues within 4.1 Å distance of the ligands are shown in stick representation. Hydrogen bonds are shown as yellow dashed lines. **d** Comparison of residues involved in ligand-binding pockets. **e–g** Diagram of ligand interactions between α_1A_AR and the ligands. Residues within 4.1 Å distance of the ligands are shown.

Extensive site-directed mutagenesis studies have identified amino acids that form the binding pocket of the α_1A_AR, including residues responsible for subtype selectivity^34–42^. Our structures largely confirm these observations. α_1A_AR structures bound to the endogenous agonist noradrenaline, selective partial agonist oxymetazoline, and selective antagonist tamsulosin are shown in Fig. 4. All three ligands form polar interactions with D106^3.32^ which is a highly conserved residue involved in ligand binding in all aminergic receptors (Fig. 4 and Supplementary Fig. 7). The binding pocket of noradrenaline is formed by residues in TMs 3, 5, 6, and 7 (Fig. 4a, e). The noradrenaline has two catechol hydroxyl groups. The para-hydroxyl forms a hydrogen bond with S188^5.42^, whereas meta-hydroxyl does not form polar interaction but is close to M292^6.55^, a unique residue among ARs (Supplementary Fig. 7). Previous mutagenesis^34–36^ and [^13^C^ε^H]methionine labeling NMR studies^37^ support the role of S188^5.42^ and M292^6.55^ in ligand binding. The chiral β-hydroxyl forms a hydrogen bond with D106^3.32^ and the amino group of the noradrenaline forms cation-π stacking with the phenyl ring of F312^7.39^ and a hydrogen bond with the backbone carbonyl of F312^7.39^. Noradrenaline forms extensive non-polar interactions with highly conserved aromatic residues among ARs, including Y184^5.38^, F288^6.51^, and F289^6.52^.

The oxymetazoline binds in a similar site (Fig. 4b, f), but the para-catechol hydroxyl is replaced by tertiary butyl, leading to the lack of polar interaction with S188^5.42^, which may account for its partial agonism in receptor activation^12,14,15^. Tertiary-butyl group forms extensive van der Waals interaction with Y184^5.38^, V185^5.39^, S188^5.42^, M292^6.55^ and the backbone carbonyl of N179^ECL2^. Position 5.39 is a valine in α_1A_AR, α_2A_AR, and β_3_AR, but is an alanine/isoleucine in other subtypes of ARs (Supplementary Fig. 7). A previous mutagenesis study showed that V185A^5.39^ and M292L^6.55^ mutations resulted in decreased oxymetazoline binding but not noradrenaline binding, and the equivalent A204V^5.39^ and L312M^6.55^ mutants of α_1B_AR increased agonist binding^35,36^. In place of the chiral β-hydroxyl and the amino group of noradrenaline, oxymetazoline has an imidazoline ring which forms polar interactions with D106^3.32^ and C110^3.36^, π-π stacking with F312^7.39^, and aromatic interactions with W285^6.48^, F288^6.51^ and Y316^7.42^. In α_1_- and α_2_ARs, C110^3.36^ and F312^7.39^ are conserved residues and involved in ligand recognition for imidazoline-type agonists^14–16^. Consistent with this result, a mutagenesis study indicated that F312A^7.39^ and F312N^7.39^ (the equivalent residues for βARs) mutations decreased oxymetazoline binding but not adrenaline binding^38^. This report also demonstrated that F308^7.35^, a residue above the F312^7.39^, influences oxymetazoline binding, even though it is too far away for a direct interaction^38^.

The antagonist tamsulosin has two aromatic groups on each side of an ethyl-aminopropyl backbone (Fig. 4c, g). Similar to agonist binding, the ethylamine group of tamsulosin forms a hydrogen bond with D106^3.32^. The ethoxyphenoxy group forms van der Waals interaction with V107^3.33^, S188^5.42^, M292^6.55^, F288^6.51^ and F289^6.52^ (Supplementary Fig. 7). Unlike agonist binding, reorientation of F312^7.39^ enlarges the binding pocket and enables the antagonist to bind towards the extracellular vestibule, which is also called an exosite or secondary binding pocket with less conserved residues compared to the orthosteric pockets^12,43^ (Fig. 4d). The sulfonamide group forms a polar interaction with the backbone carbonyl of C176^45.50^ and a non-polar interaction with F308^7.35^. The methoxybenzene group forms non-polar interactions with F86^2.64^, E87^2.65^, W102^3.28^, F312^7.39^ and the backbone carbonyl of S83^2.61^ (Fig. 4c and Supplementary Fig. 7). F86^2.64^ is a unique residue to α_1A_AR relative to other ARs and was previously identified as a determinant for the interaction of the α_1A_AR with various antagonists including HEAT and prazosin^39–41^ (Supplementary Fig. 1). In addition, another mutation study indicated that three non-conserved residues (Q177^45.51^, I178^45.52^, N179^45.53^) in ECL2 are responsible for the α_1A_AR selectivity of phentolamine and WB4101 over α_1B_AR^42^, but are not involved in binding tamsulosin.

### Ligand recognition of adrenergic receptor subtypes

**Fig. 5:**
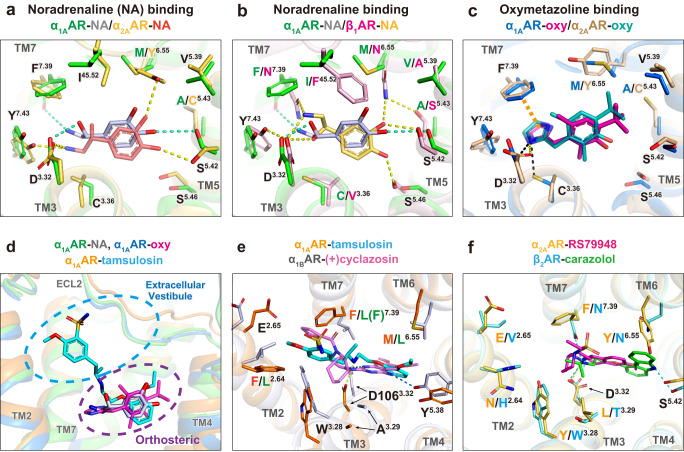
Comparisons of ligand-binding pockets for the adrenergic receptors. **a** Noradrenaline binding between α_1A_AR (green sticks with green hydrogen bonds) and α_2A_AR (yellow sticks with yellow hydrogen bonds; PDB ID: 7EJ0**)**. **b** Noradrenaline binding between α_1A_AR (green sticks) and β_1_AR (light pink with yellow hydrogen bonds; PDB ID: 7BU6). **c** Oxymetazoline binding between α_1A_AR (blue sticks with black hydrogen bonds) and α_2A_AR (wheat sticks with yellow hydrogen bonds; PDB ID: 7EJK). Aromatic interaction is shown as an orange dashed line. **d** Orthosteric and extracellular vestibule of α_1A_AR bound to noradrenaline (gray) and tamsulosin (cyan). **e** Comparison of antagonists binding to α_1A_AR (orange sticks with yellow hydrogen bonds, tamsulosin is colored cyan), and α_1B_AR (gray sticks with blue hydrogen bonds and pink ligand; PDB ID: 7B6W). Note that F^7.39^ of the α_1B_AR is mutated to L^7.39^ for stabilization. **f** Comparison of antagonists binding between α_2A_AR (light orange sticks with yellow hydrogen bonds; ligand is a colored gray stick; PDB ID: 6KUX) and β_2_AR (cyan sticks with cyan hydrogen bonds and green colored ligand; PDB ID: 2RH1).

Although all adrenergic receptors are activated by endogenous adrenaline and noradrenaline, their binding pockets are not identical. Comparisons of the key residues of the noradrenaline binding pockets in α_1A_AR, α_2A_AR^14^ and β_1_AR^11^ reveal similar but different mechanisms of noradrenaline recognition (Fig. 5a, b). Compared to M^6.55^ in α_1A_AR, Y^6.55^ in α_2A_AR (conserved in all α_2_ARs) forms a hydrogen bond with the meta-hydroxyl of the catechol ring, while the para-hydroxyl is involved in hydrogen bonds with S^5.42^ in both α_1A_AR and α_2A_AR. The rotamer of S^5.42^ differs between α_1A_AR and α_2A_AR, leading a distinct difference in the pose of the catechols. In α_1A_AR, the β-hydroxyl group interacts with D^3.32^, and the amino group forms a hydrogen bond and a cation-π interaction with F^7.39^(Figs. 4a and 5a). In contrast, only the amino group forms hydrogen bonds with both D^3.32^ and Y^7.43^ in α_2A_AR. The noradrenaline binding pose of β_1_AR is different from that of α_1A_AR (Fig. 5b). The meta-hydroxyl forms polar interaction networks with S^5.42^, S^5.43^ and N^6.55^, and the para-hydroxyl forms a hydrogen bond with S^5.46^ in β_1_AR. Previous α_1A_AR mutagenesis studies indicated that double mutation of S188A^5.42^ and S192A^5.46^ decreased agonist binding rather than S188A^5.42^ or S192A^5.46^ alone^34^. Non-aromatic N^7.39^ interacts with the amine group of noradrenaline through the polar interaction networks with D^3.32^ and Y^7.43^ in β_1_AR. Moreover, the bulkier F^45.52^ in ECL2 of β_1_AR (conserved in all βARs) forms a non-polar interaction with noradrenaline^10,11^.

We next compare the binding pose of imidazoline-type partial agonist oxymetazoline in α_1A_AR and α_2A_AR^14^ (Fig. 5c). The oxymetazoline presents high selectivity for α_1A_AR and α_2A_AR over other αARs^4^. As mentioned before, oxymetazoline replaces the para-hydroxyl with the hydrophobic tertial-butyl group which no longer forms the polar interaction but engages in hydrophobic interactions with partially conserved V^5.39^ in α_1A_AR. In α_2A_AR, oxymetazoline also does not form polar interaction with TM5, but C^5.43^ is involved in the hydrophobic interaction. This position is A189^5.43^ in α_1A_AR, while S^5.43^or C^5.43^ in other ARs (Supplementary Fig. 7). In both α_1A_AR and α_2A_AR, the imidazoline ring is stabilized by π-π stacking with F^7.39^ along with polar interaction with D^3.32^, in contrast, C^3.36^ (conserved in all αARs and V in βARs) forms weak polar interaction with the imidazoline ring only in α_1A_AR. Although most of the residues have the same orientation between noradrenaline and oxymetazoline binding in α_1A_AR, the orientation of Y^6.55^ is shifted in α_2A_AR. This Y^6.55^ is involved in G_i/o_-biased signaling over β_-_arrestin recruitment for oxymetazoline among the other agonists such as noradrenaline, brimonidine and dexmedetomidine in α_2A_AR^14^.

Compared to α_1_AR agonists, the α_1_AR antagonists have higher subtype-selectivity because they extend to the extracellular vestibule (Fig. 5d). As mentioned above, inactive α_1A_AR bound to tamsulosin reveals that unique (F86^2.64^, and M292^6.55^) and partially conserved (S83^2.61^, E87^2.65^, W102^3.28^, I178^45.52^, and F312^7.39^) residues are involved in subtype selectivity (Fig. 3c, f and Supplementary Fig. 7). Recent crystal structure of inactive α_1B_AR bound to its selective inverse agonist (+)-cyclazosin, along with the chimeric α_1B_AR-α_2C_AR mutagenesis studies indicated that non-conserved residues L^2.64^, W^3.28^, A^3.29^, V^45.52,^ and L^6.55^ are important for the selectivity in α_1B_AR^18^ (Supplementary Fig. 7). The (+)-cyclazosin is a derivative of prazosin in which piperazinyl quinazoline scaffold is introduced in a bulky cycloaliphatic group (Supplementary Fig. 1), leading to 100–1000 fold selectivity for α_1_ARs over α_2_ARs, and a slight preference for α_1B_AR over α_1A_AR. When comparing the α_1A_AR and α_1B_AR (Fig. 5e), both the tamsulosin and (+)-cyclazosin extend into the extracellular vestibule. Tamsulosin interacts with F86^2.64^ more closely than the furan group of (+)-cyclazosin, which is consistent with the binding selectivity^44^, however, a stabilizing mutation of F^7.39^ to L^7.39^ in the α_1B_AR structure might affect the (+)cyclazosin binding mode^18^. In contrast to α_1_ARs, the antagonists of α_2A_AR and β_2_AR do not interact with TM2 (Fig. 5e, f)^8,15^. The positions 2.64, 3.28, 3.29, and 45.52 are different from α_1_ARs and likely involved in the ligand selectivity. In addition, residues M^6.55^ in α_1A_AR and N^6.55^ in β_2_AR allow antagonist interactions with the extracellular side of TM6, in contrast to the bulkier Y^6.55^ in α_2A_AR.

It is known that the other α_1A_AR ligands such as A61603 (agonist) and silodosin (antagonist) have high selectivity for α_1A_AR (Supplementary Fig. 1)^37,44^. In these compounds, the phenyl rings corresponding to catechol have much bulkier substituents, suggesting that they may exhibit selectivity through interaction with α_1A_AR unique residues such as M292^6.55^, A189^5.43^ and the non-conserved residue V185^5.39^.

### Structural insight into Nb29 binding

Nb29 binds to the extracellular side of α_1A_AR which is topologically distinct from the orthosteric agonist pocket (Fig. 2). This site has been shown to bind to allosteric modulators for muscarinic receptors^45–48^. We do observe a left shift of the agonist competition binding curves in the presence of Nb29 (Fig. 1c–e); however, these experiments are complicated by the fact that Nb29 is a competitive inhibitor of the radioligand [^3^H] prazosin. In cell signaling assays, Nb29 exhibits no agonist activity on its own, has no effect on EC_50_ for oxymetazoline or noradrenaline, and slightly reduces the maximum efficacy of α_1A_AR activation (Supplementary Fig. 8a–d), suggesting that Nb29 appears to antagonize receptor activation or possibly block the ligand entry into the orthosteric pocket. It should be noted that the radioligand competition assay was performed in equilibrium and the agonists had a longer incubation time to access the orthosteric pocket than in the signaling assay. In both assays, the effects of Nb29 are larger for oxymetazoline compared with noradrenaline, which is consistent with Nb29’s binding selectivity towards the oxymetazoline-bound state of the α_1A_AR in the titration assay (Fig. 1b–e and Supplementary Fig. 8). Thus, Nb29 might be considered a weak positive allosteric modulator (PAM) or a neutral allosteric modulator, given that it binds to a known allosteric binding pocket in other GPCRs.

**Fig. 6:**
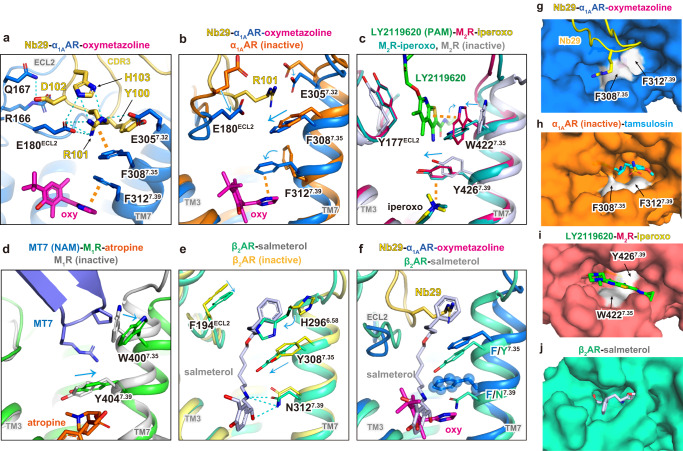
Structural comparison of the Nb29 binding site. Nb29 binding sites of (**a**) Nb29-α_1A_AR-oxymetazoline; **b** Nb29-α_1A_AR-oxymetazoline and inactive α_1A_AR; **c** LY2119620-M_2_R-iperoxo (PDB ID: 4MQT), M_2_R-iperoxo (PDB ID: 4MQS) and inactive M_2_R (PDB ID: 3UON); **d** MT7-M_1_R-atropine (PDB ID: 6WJC) and inactive M_1_R (PDB ID: 5CXV); **e** β_2_AR-salmeterol (PDB ID: 6MXT) and inactive β_2_AR (PDB ID: 2RH1); **f** Nb29-α_1A_AR-oxymetazoline and β_2_AR-salmeterol (PDB ID: 6MXT). Polar and aromatic interactions are shown as cyan and orange dashed lines, respectively. Conformational changes are shown with cyan arrows. **g–j** Surface representations of top views of Nb29-α_1A_AR-oxymetazoline, inactive α_1A_AR-tamsulosin, LY2119620-M_2_R-iperoxo (PDB ID: 4MQT) and β_2_AR-salmeterol (PDB ID: 6MXT).

The Nb29 binding interactions are almost identical in oxymetazoline- and noradrenaline-bound Nb29-α_1A_AR complexes, but the map resolution is relatively poor in the noradrenaline-bound state (Fig. 1 and Supplementary Figs. 3 and 4). Thus, we used the oxymetazoline-bound state for structural analysis. Nanobodies consist of three complementarity-determining regions (CDRs). The relatively long CDR3 interacts with a broad range of residues from ECL2 and with E305^7.32^ at the top of TM7 (Supplementary Figs. 9a–c and 10a, b). Among them, seven amino acids (R166, Q167, E171, T174, Q177, N179, and E305^7.32^) are non-conserved residues in α_1_AR subtypes, suggesting that these residues are involved in nanobody specificity (Supplementary Figs. 7, 9a–c). Nb29 binding also stabilized the polar interaction network between ECL2 and R96^3.22^, which is not observed in the inactive α_1A_AR structure without Nb29 (Supplementary Fig. 9d–f). Four residues of CDR3 (Y100, R101, D102 and H103) bind to the extracellular vestibule from the agonist-binding pocket (Fig. 6a). R101 of Nb29 forms charge networks with E180^ECL2^ and E305^7.32^, and a cation-π interaction with F308^7.35^ of α_1A_AR, stabilizing the inward conformation of TM7 (Fig. 6a, b). As mentioned above, F308^7.35^ and the close-lid conformation of F^7.39^ is important for the agonist binding in αARs (Fig. 4)^38^, the π-π stacking of oxymetazoline with F312^7.39^ might contribute to the Nb29 binding selectivity compared with the cation-π stacking of noradrenaline with F312^7.39^.

The residues at position 7.35 have also been identified as critical residues for both PAM and negative allosteric modulator (NAM) binding of muscarinic acetylcholine receptors (MRs) by stabilizing the extracellular side of TM7 either in inward or outward conformations, respectively^45,46^. The LY2119620, a PAM for M_2_R, binds to the extracellular vestibule and changes the conformation of W422^7.35^ by an aromatic stacking, whereas those of the other residues are almost identical between the LY2119620-M_2_R-iperoxo and M_2_R-iperoxo complexes (Fig. 6c and Supplementary Fig. 10c, d)^45^. In contrast to this PAM, a peptide toxin MT7, the NAM for M_1_R activation, stabilizes the outward displacement of TMs 6 and 7 through interactions with W400^7.35^^46^ (Fig. 6d and Supplementary Fig. 10e, f). Moreover, a mutagenesis study indicated that F330^7.35^ in α_1B_AR is involved in the allosteric binding for conotoxin ρ-TIA, a selective NAM for α_1B_AR among α_1_ARs^49^. The Nb29 binding site also overlaps with the aryloxyalkyl tail of the selective agonist salmeterol binding in β_2_AR (Fig. 6e, f)^12^. The smaller N^7.39^ in β_2_AR enables the salmeterol to extend into the extracellular vestibule to make aromatic interactions with F194^ECL2^, H296^6.58^ and Y308^7.35^, which are unique to β_2_AR (Supplementary Fig. 7). Among aminergic receptors, the aromatic amino acid at position 7.39 is observed in αARs (F^7.39^), muscarinic receptors (Y^7.39^) and histamine H_3_ and H_4_ receptors (F^7.39^)^12,43,45,50^. The selective agonists targeting the extracellular vestibule of αARs are limited compared to other GPCRs such as βARs and muscarinic receptors^12,13,43,50^ (Fig. 6g–j and Supplementary Fig. 1).

Subsequently, we compared the nanobody binding site with available class A GPCR structures in complex with extracellular nanobodies or antibody Fab fragments (Supplementary Fig. 10). Consistent with the review for GPCR antibodies^19^, peptide-binding GPCRs are more frequently targeted by antibodies because they have relatively large binding pockets compared with the small-molecule binding GPCRs such as aminergic GPCRs. Only two structures in complex with extracellular nanobodies have been reported in class A GPCRs, which are the apelin receptor (APJ)^24^ and the orexin receptor 2 (OX_2_R)^51^, although more structures of the intracellular binding nanobodies have been published for stabilizing the GPCR active conformations as G protein mimetics, such as Nb9-8 for M_2_R^10,20,45^ (Supplementary Fig. 10c–j). Anti-APJ nanobody JN241 antagonizes APJ through extensive interactions with extracellular loops of APJ and the insertion of CDR3 into the peptide-binding site^24^, whereas anti-OX_2_R nanobody Sb51 is positioned above the small-molecule agonist and partially overlaps with natural-peptide orexin B binding site^51^. In contrast to nanobodies, conventional antibodies and Fab fragments consist of heavy (CDRs H1-3) and light (CDRs L1-3) chains (Supplementary Fig. 10k–r). The antibody for protease-activated receptor 2 (PAR2) behaved as an antagonist by blocking ligand access from the extracellular region by both heavy and light chains (H1, H3, L2, and L3)^26^. In the angiotensin II type 2 receptor (AT2)^23^, EP4^22^, and D_2_R structures^25^, their antibodies allosterically enhance the ligand binding. The antibody for AT2 bound to the ECL1 and β-hairpin motif of ECL2 to stabilize the peptide agonist binding pocket, while the antibody for EP4 stabilizes the occluded β-hairpin of ECL2, leading to enhanced antagonist binding. In the D_2_R-Fab3089 structure, the CDR-H2 stabilizes the antagonist spiperone which binds toward the extracellular vestibule.

Taken together, the Nb29 structure provides insights into allosteric binding and antibody recognition for α_1A_AR. Nb29 covers the extracellular surface of α_1A_AR like anti-APJ nanobody JN241 and anti-PAR2 Fab, whereas the CDR3 loop of Nb29 binds to a similar site of PAM for M_2_R (Fig. 6 and Supplementary Fig. 10). It should be noted that nanobodies are amenable to optimization due to the single variable domain. For example, the G protein-mimicking nanobody for β_2_AR was optimized by directed evolution to increase its affinity to the receptor^10^; and the APJ nanobody antagonist JN241was rationally engineered into an APJ agonist by structure-guided site-directed mutation of CDR3^24^.

### Selectivity of G protein interactions with adrenergic receptors

**Fig. 7:**
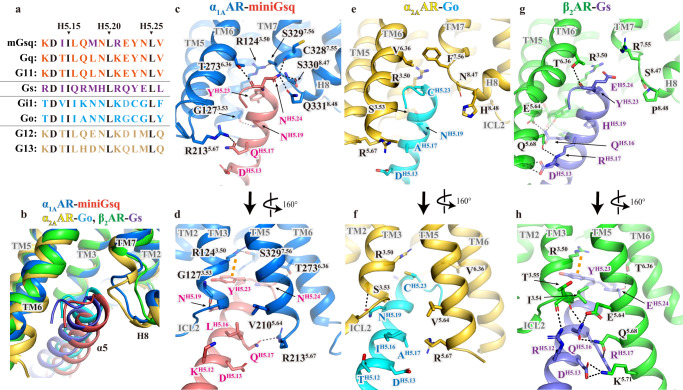
Comparison of the receptor-G protein binding interfaces of the adrenergic receptor subtypes. **a** Sequence alignments of the carboxyl-terminal α5-helix of G proteins subtypes. **b** Superimposition of the binding interfaces of α_1A_AR-miniGsq complexes with α_2A_AR-Go (PDB ID: 7EJ0) and β_2_AR -Gs (PDB ID: 3SN6) complexes. The receptors are used for alignment. The proteins are colored as follows: α_1A_AR (cobalt), miniGsq (pink), α_2A_AR (yellow), Go (cyan), β_2_AR (green), and Gs (blue purple). Detailed polar interactions and the equivalent residues of α_1A_AR-miniGsq (**c**, **d**), α_2A_AR-Go (**e**, **f**), and β_2_AR -Gs (**g**, **h**). The polar interactions are shown as black dashed lines.

Noradrenaline- and oxymetazoline-bound α_1A_AR-miniGsq complexes are almost identical at the interfaces with miniGsq, as observed in α_2A_AR-Go complexes with different agonists^14^. Thus, we used the oxymetazoline-bound active state for structural analysis. Of the 15 residues at the carboxyl-terminal α5-helix of miniGsq (Fig. 7a)^29^, seven residues are specific to Gq, including K^H5.12^, L^H5.16^, Q^H5.17^, N^H5.19^, E ^H5.22^, N^H5.24^, and V^H5.26^ (superscript, CGN G protein numbering system^52^); five residues [D^H5.13^, I^H5.15^, L^H5.20^, Y^H5.23^, and L^H5.25^] are conserved between Gs and Gq, and the other three residues [I^H5.14^, M^H5.18^ and R^H5.21^] are located on the opposite side of the interface. When comparing the interactions of α_1A_AR-miniGsq complexes with α_2A_AR-Go^14^ and β_2_AR-Gs complexes^9^, the α5-helix of miniGsq is slightly shifted towards helix 8 of α_1A_AR (Fig. 7b–h). In the α_1A_AR-miniGsq complex, α_1A_AR forms six hydrogen bonds with the residues corresponding to Gq: between R213^5.67^ and Q^H5.17^, between the backbone carbonyl of G127^3.53^ and N^H5.19^, between T273^6.36^ and the backbone carbonyl of N^H5.24^; the side chain of N^H5.24^ forms hydrogen bond networks with the backbone carbonyl of C328^7.55^, the backbone carbonyl of S330^8.47^, and the side chain of Q331^8.48^ (Fig. 7c, d). These residues of α_1A_AR are not conserved in α_2_ARs and βARs (Supplementary Fig. 11). The R^3.50^ forms cation-π stacking interaction with Y^H5.23^, which is also observed in the β_2_AR-Gs complex, but not in α_2A_AR-Go complex as this position is C^H5.23^ in Go protein (Fig. 7c–h). In the α_2A_AR-Go complex, polar interactions are observed between S^3.53^ and N^H5.19^, and hydrophobic interactions are predominant (Fig. 7e, f). In the β_2_AR-Gs complex, one side of the α5-helix of the Gs protein forms a cluster of hydrogen-bond interactions with TM3 (I^3.54^ and T^3.55^) and TM5 (E^5.64^, Q^5.68^, and K^5.71^), leading the α5-helix to shift towards TM5 (Fig. 7b, g, h). In addition to α5-helix, the N-terminal helix of G_α_ subunits are also involved in GPCR-G protein interactions^9,14^. The previous study indicates that polybasic cluster at the C terminus of M_1_R, which is conserved among most G_q/11_-coupling GPCRs, interacts with the G-protein Gα_11_/β interface^53^. However, our structure lacks these regions and there are currently no other active structures of α_1_ARs. While α_1A_AR is predominantly coupled to G_q/11_ proteins, a few studies have identified G_12/13_^54^ and β-arrestin signaling pathways^2,55^. Further studies will be required to better understand the mechanism of activation and selectivity of α_1_AR signaling.

## Discussion

Here, we present three cryo-EM structures of α_1A_AR in both active and inactive states. These structures reveal several structural aspects of α_1A_AR. The ligand-binding modes of the endogenous agonist noradrenaline and the imidazoline-type agonist oxymetazoline demonstrated a key aromatic interaction involving F312^7.39^, which is conserved in αARs, and distinct ligand recognition by the unique residue M292^6.55^. The inactive α_1A_AR bound to tamsulosin reveals the subtype selectivity of antagonist binding pockets involving F86^2.64^. Our results also provide structural insights into nanobody recognition for α_1A_AR. Nb29 binds to the extracellular vestibule of α_1A_AR and the cationic residue of CDR3 stabilizes F308^7.35^ which is the equivalent binding site of the positive allosteric modulator for M_2_R. Finally, our active α_1A_AR structures provide insight into G protein binding selectivity by comparisons with α_1A_AR-miniGsq, α_2A_AR-Go, and β_2_AR-Gs structures. Together with our α_1A_AR structures and previously published structures of α_2_ARs and βARs, the active and inactive structures of the major subtypes of the adrenergic receptor family have been determined. These results should facilitate the design of more selective and effective therapeutic drugs targeting both orthosteric and allosteric sites in this receptor family.

## Methods

### Construction

C-terminus truncated human α_1A_AR (residues 1–370, full length: 466) was modified by mutation of the N-linked glycosylation sites to glutamine (N7Q, N13Q and N22Q), N-terminal addition of the hemagglutinin signal peptide, FLAG-tag epitope, and C-terminal addition of the 8 × His-tag. For the active α_1A_AR structure, residues 223–261 of intracellular loop 3 were replaced with minimal T4L^28^. For the inactive α_1A_AR structure, we swapped residues from C205^5.59^ to G275^6.38^ with residues from T247^5.59^ to L277^6.38^ of kOR^21^. In addition, we introduced two thermostabilizing point mutations S113R^3.39^ and M115W^3.41^ which were previously used for structure determination of several inactive-state GPCRs^22,25,31^. The primers used in this study were obtained from Rui Biotech (Beijing, China), Xianghong Biotech (Beijing, China) or Genewiz (Beijing, China). The DNA sequencing analysis was performed at the Rui Biotech (Beijing, China).

### Expression and purification of α_1A_AR

Recombinant baculovirus was generated using the Bac-to-Bac Baculovirus Expression System (Thermo Fisher Scientific). *Sf9* insect cells at a cell density of 4 × 10^6^ cells/ml in ESF-921 insect media (Expressions Systems) with 20 μg/ml gentamycin and 1 μM ligand were infected with baculovirus and shaken at 27 °C for 2 days. Cells were harvested by centrifugation and stored at −80 °C until use.

To purify the protein, receptor-expressing *Sf9* cells were lysed by resuspension in a buffer containing 20 mM Tris-HCI pH 7.5, 1 mM EDTA, 10 µM ligand, 160 μg/ml benzamidine, 100 μg/ml leupeptin. The cell membranes were centrifuged at 10,000 *g* for 20 min at 4 °C. Receptor was extracted from cell membranes with solubilization buffer of 20 mM HEPES pH 7.5, 1% dodecyl maltoside (DDM), 0.03% cholesterol hemisuccinate (CHS), 0.2% Na cholate, 750 mM NaCl, and 30% glycerol. Iodoacetamide (2 mg ml^−1^) was added to block reactive cysteines at this stage. Nickel-NTA agarose was added to the solubilized receptor without prior centrifugation. After stirring for 2 h at 4 °C, receptor-bound nickel resin was washed and poured into a glass column, and the receptor was eluted in 20 mM HEPES pH 7.5, 0.1% DDM, 0.03% CHS, 0.02% Na cholate, 750 mM NaCl, 10 µM ligand and 250 mM imidazole. Nickel resin-purified receptor was bound to M1-Flag affinity resin with 2 mM CaCl_2_. Following extensive washing, detergent was gradually exchanged from DDM to 0.01% lauryl maltose neopentyl glycol (MNG). The receptor was eluted with 0.2 mg/ml Flag peptide and 5 mM EDTA and further purified by size exclusion chromatography (SEC) on a Sephadex S200 increase column (Cytiva) in a buffer of 20 mM HEPES pH 7.5, 0.01% MNG, 0.001% CHS, 100 mM NaCl and 10 µM ligand. The purified receptor was concentrated with a 50 kDa cutoff Amicon centrifugal filters (Millipore).

### Discovery of the conformationally selective α_1A_AR nanobody

The synthetic nanobody library displayed on the surface of BJ5465 yeast strain was obtained from Drs. A. C. Kruse (Harvard University) and A. Manglik (University of California San Francisco)^27^. The yeast cells were recovered in tryptophane dropout (-Trp) medium [prepared by Yeast Synthetic Drop-out Medium Supplements without tryptophane (sigma) and Yeast Nitrogen Base without amino acids (BD Difco) at pH 6.0] with 2% (w/w) glucose at 30 °C, and the nanobody was induced by -Trp medium with 2% (w/w) galactose at 25 °C. Expression levels of nanobody were estimated by staining with anti-HA antibody (Cell Signaling Tech) and analyzing by flow cytometry with an Accuri C6 (BD Biosciences)

Induced yeast cells were washed and resuspended in a selection buffer (20 mM HEPES pH 7.5, 150 mM NaCl, 0.05% MNG, 0.005% CHS, 2.8 mM CaCl_2_, 0.1% (w/v) bovine serum albumin and 5 mM maltose). Nanobody clones against the purified FLAG-tagged α_1A_AR (C-terminus truncated after residue 370) bound to oxymetazoline were enriched by two rounds of magnetic-activated cell sorting (MACS) and four rounds of fluorescence-activated cell sorting (FACS) (See Fig. 1a). For the first round of the MACS, 5 × 10^9^ yeast cells were precleared by incubating with Alexa Fluor-647 conjugated anti-FLAG M1 antibody (M1-647, prepared by anti-FLAG M1 antibody and Alexa Fluor 647-NHS ester) and anti-Alexa Fluor-647 microbeads (Miltenyi) and passed LD column (Miltenyi) to remove nonspecific nanobody. Flowed-through yeast cells were washed with the selection buffer, then incubated with 0.2 µM α_1A_AR bound to oxymetazoline, the antibody and the microbeads. After incubation at 4 °C for 30 min, yeast cells were loaded on the LD column, washed with the selection buffer and the eluted yeast cells (3.4 × 10^6^ cells) by plunger were expanded and used in a subsequent round of MACS. The second round of MACS was performed similarly to the first, but beginning with 4 × 10^8^ yeast cells and using biotin-conjugated anti-FLAG M1 antibody Fab fragment (anti-FLAG M1 antibody was digested by papain then labeled with biotin-NHS ester), streptavidin microbeads (Miltenyi) and LS column (Miltenyi) were used, and 5 × 10^6^ yeast cells were eluted.

Subsequently, we performed four rounds of FACS by FACSAria II (BD Biosciences) (See Supplementary Fig. 2). For the selection rounds 3 and 6, yeast cells were stained with Alexa Fluor-488 or −647 conjugated anti-HA antibody (Cell Signaling Tech) and 0.1 µM FLAG-tagged α_1A_AR with anti-FLAG M1-647 or −488. For the selection rounds 4 and 5, in order to enrich for conformational selective nanobodies, yeast cells were stained with two different populations of α_1A_AR labeled with anti-FLAG M1-488 and −647 fluorophores, one bound with oxymetazoline and another bound to tamsulosin. Staining yeast cells for each round of FACS experiments were the following; 5 × 10^7^ cells for round 3 and 1 × 10^7^ cells for rounds 4–6. After round 6, the sorted yeast cells were diluted and plated on -Trp agar plates. Single clones were sequenced and cloned into the periplasmic expression vector pET26b, containing an N-terminal pelB signal sequence and a C-terminal histidine tag, and transformed into BL21(DE3) *Escherichia coli*. Cells were induced in Terrific Broth medium with 2 mM MgCl_2_, 0.1% glucose and 50 µg/ml kanamycin at an OD600 of 0.7 with 1 mM IPTG and incubated with shaking at 25 °C for 20 h. Periplasmic protein was obtained by osmotic shock in a buffer containing 0.2 M Tris pH 8.0, 0.5 mM EDTA and 0.5 M sucrose at 4 °C for 1 h, then diluted 4 times and incubated for another one hour. The lysate was centrifuged and the supernatant was purified by Ni-NTA resin and size-exclusion chromatography.

For the on-yeast titration assay, Nb29-displayed yeast cells were stained with the Alexa Fluor-647 conjugated anti-HA antibody and several concentrations of purified α_1A_AR fused at the C-terminus to an enhanced green fluorescent protein in the presence or absence of 500 μM ligands in the selection buffer. Yeast cells were analyzed by Accuri C6 and the ratio of double-positive yeast cells among anti-HA positive cells was calculated.

### Purification of the Nb29-α_1A_AR-miniGsq and the α_1A_AR-Nb6 complexes

We modified the expression and purification method of miniGsq from the previous report^29,56^, in which miniGsq was used instead of miniGq for the expression in *Escherichia coli*. The pET21a plasmid encoding miniGsq and N-terminal histidine tag was transformed into BL21(DE3) *Escherichia coli*. Cells were induced in Terrific Broth medium at an OD600 of 0.6 with 1 mM IPTG and incubated with shaking at 25 °C for 20 h. Cells were harvested and lysed by sonication in a buffer containing 40 mM Hepes pH 7.5, 100 mM NaCl, 10 mM imidazole, 10% glycerol, 5 mM MgCl_2_, 50 μM guanosine diphosphate (GDP), 100 μM dithiothreitol, 160 μg/ml benzamidine, and 100 μg/ml leupeptin. The lysate was centrifuged at 10,000 g for 20 min at 4 °C and the supernatant was immobilized by Ni-NTA resin. The eluate was further purified by size-exclusion chromatography in a buffer containing 10 mM HEPES pH 7.5, 100 mM NaCl, 1 mM MgCl_2_, 10 μM GDP and 100 μM tris(2-carboxyethyl)phosphine (TCEP).

The Nb29-α_1A_AR-miniGsq complex was prepared by mixing with the purified α_1A_AR bound the agonist (oxymetazoline or noradrenaline), Nb29 and miniGsq in a 1:1.2:1.2 molar ratio and supplemented with apyrase. After incubation for 2 h at room temperature, the mixtures were purified by size exclusion in SEC buffer containing 20 mM HEPES pH 7.5, 100 mM NaCl, 0.002% MNG, 0.0002% CHS, 2 mM MgCl_2_, 10 μM agonist and 100 μM TCEP. Fractions containing the complex were concentrated to 5–10 mg/ml using a 50 kDa molecular weight cutoff Amicon Ultra concentrator. For the α_1A_AR-Nb6 complexes, Nb6 from gene synthesis^33^ was prepared as the same as nanobody purification described above. Purified α_1A_AR-kOR swap construct and Nb6 were mixed in a 1:1.5 molar ratio in the presence of tamsulosin and incubated on ice for 30 min. The mixture was further purified by size exclusion in SEC buffer containing 20 mM HEPES pH 7.5, 100 mM NaCl, 0.002% MNG, 0.0002% CHS, 2 mM MgCl_2,_ and 10 μM ligand. Fractions containing the complex were concentrated using a 50 kDa molecular weight cutoff Amicon Ultra concentrator.

### Ligand binding assay

Ligand binding assays were performed with *Sf9* cell membrane or purified α_1A_AR-bound M1-Flag affinity resin. Receptor-expressing cells were harvested and homogenized in a binding buffer containing 20 mM Tris–HCl (pH 7.5) and 100 mM NaCl. After centrifugation, the pellet was homogenized in binding buffer and used as the membrane fraction in binding assays. The purified α_1A_AR-bound M1-Flag affinity resin was resuspended in binding buffer containing 20 mM HEPES pH 7.5, 100 mM NaCl, 0.01% MNG, 0.001% CHS and 2 mM CaCl_2_.

Radioligand binding assay was performed using [^3^H]prazosin (PerkinElmer). Receptors were incubated for 1 h at room temperature with various concentrations of ligand in a total volume of 100 µl. After the reaction, the mixture was trapped on Whatman GF/B glass filters. Bound and free radioligands were separated by washing with ice-cold binding buffer. Radioactivity was measured on a MicroBeta2 liquid scintillation counter (PerkinElmer). All binding assay measurements were analyzed using the Prism 9 software (GraphPad).

### Cryo-EM sample preparation and data acquisition

Two of the Nb29–α_1A_AR-miniGsq complexes were collected cryo-EM data at Tsinghua University, and the α_1A_AR-Nb6 complex was performed data acquisition by the Shuimu Bioscience (Beijing). The purified protein complexes were concentrated to 5–10 mg/mL. 4 μl of sample were applied to the glow-discharged holey carbon grids (Au R1.2/1.3, 300 mesh) purchased from Quantifoil for the Nb29-α_1A_AR-miniGsq complexes, and from Zhongjingkeyi Technology (Beijing) for the α_1A_AR-Nb6 complex. The grids were blotted for 3.0 s and flash-frozen in liquid ethane cooled by liquid nitrogen with Vitrobot (Mark IV, Thermo Fisher Scientific) before being transferred to a 300 kV Titan Krios microscope equipped with Gatan K3 Summit detector and a GIF Quantum energy filter (slit width 20 eV) or Falcon-4 detector and no energy filter. AutoEMation was used for the fully automated data collection in Tsinghua University^57^. The total dose of each stack was about 50 e^−^/Å^2^. All frames in each stack were aligned and summed using the whole-image motion correction program MotionCor2^58^ and binned to a pixel size of 1.083 Å/1.098 Å/0.860 Å for Nb29-α_1A_AR-miniGsq bound to oxymetazoline/ Nb29-α_1A_AR-miniGsq bound to noradrenaline/α_1A_AR-Nb6 bound to tamsulosin datasets, respectively. The defocus value of each image, which was set from −1.3 to −1.8 μm during data collection, was determined by Gctf^59^.

### Cryo-EM data processing

For Nb29-α_1A_AR-miniGsq bound to oxymetazoline/ Nb29-α_1A_AR-miniGsq bound to noradrenaline/ α_1A_AR-Nb6 bound to tamsulosin datasets, 700/1099/1911 dose-weighted micrographs were imported into cryoSPARC and CTF parameters were estimated by using patch-CTF, respectively. 1,161,201/2,597,118/2,334,500 particles picked by blob picker or template picker were extracted and subjected to 2D classification. 697,792/774,350/885,066 particles remained to generate the initial model by Ab-Initio Reconstruction and perform the following iterative rounds of heterogeneous refinement. After non-uniform refinement and local refinement, 359,833/ 393,438/ 285,284 particles yield the maps which reached the resolutions at 2.92 Å/3.52 Å/3.35 Å.

### Model building and refinement

The atomic coordinate of the Nb29-α_1A_AR-miniGsq and the α_1A_AR-Nb6 complexes was generated by combining homology modeling and de novo model building. An initial structure model for the active α_1A_AR was predicted by the homology model from GPCRdb (gpcrdb.org)^60^, α_1A_AR-kOR was generated by AlphaFold2^61^ and the structure model of Nb29 was predicted by the homology model from swiss-model^62^. The initial models of miniGsq and Nb6 were imported from miniGs (PDB code: 5G53)^29^ and Nb6 (PDB code: 6VI4)^33^, respectively. The cryo-EM model was docked into the electron microscopy density map using UCSF Chimera^63^, followed by iterative manual adjustment and rebuilding in PHENIX^64^ and COOT^65^. The structures were refined against the corresponding map using PHENIX and COOT in real space with secondary structure and geometry restraints. Figures were created using the PyMOL Molecular Graphics System v.2.4.0 (Schrӧdinger, LLC), UCSF Chimera and the UCSF Chimera X1.3 package.

### Glo-sensor signaling assay

To determine the signaling profile of Nb29 against α_1A_AR, we used a cAMP Glo-sensor kit (Promega) with an engineered Gsq protein in which 15 residues at the C-terminus of Gs protein were replaced with those of Gq protein. The Gsq protein could be activated by the α_1A_AR and stimulates intracellular cAMP production. In brief, pGloSensor™−22F plasmid, receptor plasmid, and Gsq plasmid were transfected into HEK293T cells. At 24 h after transfection, the cells were switched into a CO_2_-Independent Medium (Gibco) and incubated with GloSensor^TM^ cAMP reagent. The mixture was then transferred to a 96-well white plate. The 96-well plate is placed at 37 °C in the dark for 1 h, then placed at room temperature in the dark for 1 h before use. The luminescence signal was measured by Ensight^TM^ plate reader (PerkinElmer) around 10–15 min after the addition of the agonist and/or Nb29. The result curves were calculated and fitted by GraphPad Prism 9.

### Reporting summary

Further information on research design is available in the Nature Portfolio Reporting Summary linked to this article.

## Supplementary information

Supplementary Information

Peer Review File

Reporting Summary

## Data Availability

Atomic coordinates and cryo-EM maps for the reported structures were deposited in the Protein Data Bank under accession codes 7YM8 (Nb29-α_1A_AR-miniGsq bound to oxymetazoline), 7YMH (Nb29-α_1A_AR-miniGsq bound to noradrenaline), and 7YMJ (α_1A_AR-Nb6 bound to tamsulosin), and in the Electron Microscopy Data Bank under accession codes EMDB-33924 (Nb29-α_1A_AR-miniGsq bound to oxymetazoline), EMDB-33928 (Nb29-α_1A_AR-miniGsq bound to noradrenaline), and EMDB-33930 (α_1A_AR-Nb6 bound to tamsulosin), respectively. Previously published structures can be accessed via accession codes: 5G53, 6VI4, 7EJ0, 7BU6, 7EJK, 7B6W, 2RH1, 6KUX, 4MQT, 3UON, 6WJC, 5CXV, 6MXT, 7EJ0, 7UL2, 6WJC, 6KNM, 7L1V, 5YWY, 7DFP. Source data are provided with this paper.

## Source data

Source Data

## References

[1] Schwinn DA (1990). Molecular cloning and expression of the cDNA for a novel alpha 1-adrenergic receptor subtype. J. Biol. Chem..

[2] Perez DM (2021). Current developments on the role of α(1)-adrenergic receptors in cognition, cardioprotection, and metabolism. Front. Cell Dev. Biol..

[3] Docherty JR (2010). Subtypes of functional alpha1-adrenoceptor. Cell Mol. Life Sci..

[4] Haenisch B (2010). Alpha-adrenoceptor agonistic activity of oxymetazoline and xylometazoline. Fundam. Clin. Pharmacol..

[5] Hollingsworth JM, Wilt TJ (2014). Lower urinary tract symptoms in men. BMJ.

[6] Paiva HS, Filho IJZ, Cais C (2021). Using prazosin to treat posttraumatic stress disorder and associations: a systematic review. Psychiatry Investig..

[7] Li S (2022). Inpatient administration of alpha-1-adrenergic receptor blocking agents reduces mortality in male COVID-19 patients. Front. Med..

[8] Cherezov, V. et al. High-resolution crystal structure of an engineered human beta2-adrenergic G protein-coupled receptor. *Science*. **318** 1258–1265 (2007). 10.1126/science.1150577 PMC2583103 17962520

[9] Rasmussen SG (2011). Crystal structure of the beta2 adrenergic receptor-Gs protein complex. Nature.

[10] Ring AM (2013). Adrenaline-activated structure of β2-adrenoceptor stabilized by an engineered nanobody. Nature.

[11] Xu X (2021). Binding pathway determines norepinephrine selectivity for the human β(1)AR over β(2)AR. Cell Res.

[12] Masureel M (2018). Structural insights into binding specificity, efficacy and bias of a β(2)AR partial agonist. Nat. Chem. Biol..

[13] Nagiri C (2021). Cryo-EM structure of the β3-adrenergic receptor reveals the molecular basis of subtype selectivity. Mol. Cell.

[14] Xu J (2022). Structural insights into ligand recognition, activation, and signaling of the α(2A) adrenergic receptor. Sci. Adv..

[15] Qu L (2019). Structural basis of the diversity of adrenergic receptors. Cell Rep..

[16] Yuan, D. et al. Activation of the alpha2B adrenoceptor by the sedative sympatholytic dexmedetomidine. *Nat. Chem. Biol.* **16**, 507–512 (2020). 10.1038/s41589-020-0492-2 32152538

[17] Chen X (2019). Molecular mechanism for ligand recognition and subtype selectivity of α(2C) adrenergic receptor. Cell Rep..

[18] Deluigi M (2022). Crystal structure of the α(1B)-adrenergic receptor reveals molecular determinants of selective ligand recognition. Nat. Commun..

[19] Hutchings CJ (2017). Opportunities for therapeutic antibodies directed at G-protein-coupled receptors. Nat. Rev. Drug Discov..

[20] Manglik A, Kobilka BK, Steyaert J (2017). Nanobodies to study G protein-coupled receptor structure and function. Annu. Rev. Pharmacol. Toxicol..

[21] Robertson, M. J. et al. Structure determination of inactive-state GPCRs with a universal nanobody. *bioRxiv*10.1101/2021.11.02.466983 (2021). 10.1038/s41594-022-00859-8 PMC12014012 36396979

[22] Toyoda Y (2019). Ligand binding to human prostaglandin E receptor EP(4) at the lipid-bilayer interface. Nat. Chem. Biol..

[23] Asada H (2018). Crystal structure of the human angiotensin II type 2 receptor bound to an angiotensin II analog. Nat. Struct. Mol. Biol..

[24] Ma Y (2020). Structure-guided discovery of a single-domain antibody agonist against human apelin receptor. Sci. Adv..

[25] Im D (2020). Structure of the dopamine D(2) receptor in complex with the antipsychotic drug spiperone. Nat. Commun..

[26] Cheng RKY (2017). Structural insight into allosteric modulation of protease-activated receptor 2. Nature.

[27] McMahon C (2018). Yeast surface display platform for rapid discovery of conformationally selective nanobodies. Nat. Struct. Mol. Biol..

[28] Thorsen TS (2014). Modified T4 lysozyme fusion proteins facilitate G protein-coupled receptor crystallogenesis. Structure.

[29] Nehmé R (2017). Mini-G proteins: Novel tools for studying GPCRs in their active conformation. PLoS One.

[30] Ballesteros, J. A. & Weinstein H. Integrated methods for the construction of three dimentional models and computational probing of structure-function relations in G-protein coupled receptors. *Methods Neurosci.* **25**, 366–428 (1995).

[31] Yasuda S (2017). Hot-spot residues to be mutated common in G protein-coupled receptors of class A: identification of thermostabilizing mutations followed by determination of three-dimensional structures for two example receptors. J. Phys. Chem. B.

[32] Roth CB, Hanson MA, Stevens RC (2008). Stabilization of the human beta2-adrenergic receptor TM4-TM3-TM5 helix interface by mutagenesis of Glu122(3.41), a critical residue in GPCR structure. J. Mol. Biol..

[33] Che T (2020). Nanobody-enabled monitoring of kappa opioid receptor states. Nat. Commun..

[34] Hwa J, Perez DM (1996). The unique nature of the serine interactions for alpha 1-adrenergic receptor agonist binding and activation. J. Biol. Chem..

[35] Hwa J, Graham RM, Perez DM (1995). Identification of critical determinants of alpha 1-adrenergic receptor subtype selective agonist binding. J. Biol. Chem..

[36] Hwa J, Graham RM, Perez DM (1996). Chimeras of alpha1-adrenergic receptor subtypes identify critical residues that modulate active state isomerization. J. Biol. Chem..

[37] Wu FJ (2020). Probing the correlation between ligand efficacy and conformational diversity at the α(1A)-adrenoreceptor reveals allosteric coupling of its microswitches. J. Biol. Chem..

[38] Waugh DJ (2001). Phe-308 and Phe-312 in transmembrane domain 7 are major sites of alpha 1-adrenergic receptor antagonist binding. Imidazoline agonists bind like antagonists. J. Biol. Chem..

[39] Maïga A (2014). Molecular exploration of the α(1A)-adrenoceptor orthosteric site: binding site definition for epinephrine, HEAT and prazosin. FEBS Lett..

[40] Hamaguchi N (1996). Phenylalanine in the second membrane-spanning domain of alpha 1A-adrenergic receptor determines subtype selectivity of dihydropyridine antagonists. Biochemistry.

[41] Hamaguchi N (1998). Alpha 1-adrenergic receptor subtype determinants for 4-piperidyl oxazole antagonists. Biochemistry.

[42] Zhao MM, Hwa J, Perez DM (1996). Identification of critical extracellular loop residues involved in alpha 1-adrenergic receptor subtype-selective antagonist binding. Mol. Pharmacol..

[43] Egyed A, Kiss DJ, Keserű GM (2022). The impact of the secondary binding pocket on the pharmacology of class A GPCRs. Front. Pharmacol..

[44] Quaresma BMCS (2019). Revisiting the pharmacodynamic uroselectivity of <em>α</em>1-adrenergic receptor antagonists. J. Pharmacol. Exp. Ther..

[45] Kruse AC (2013). Activation and allosteric modulation of a muscarinic acetylcholine receptor. Nature.

[46] Maeda S (2020). Structure and selectivity engineering of the M(1) muscarinic receptor toxin complex. Science.

[47] May LT (2007). Allosteric modulation of G protein-coupled receptors. Annu. Rev. Pharmacol. Toxicol..

[48] Thal DM (2018). Structural insights into G-protein-coupled receptor allostery. Nature.

[49] Ragnarsson L (2013). Conopeptide ρ-TIA defines a new allosteric site on the extracellular surface of the α1B-adrenoceptor. J. Biol. Chem..

[50] Vass M (2019). Aminergic GPCR-ligand interactions: a chemical and structural map of receptor mutation data. J. Med. Chem..

[51] Hong C (2021). Structures of active-state orexin receptor 2 rationalize peptide and small-molecule agonist recognition and receptor activation. Nat. Commun..

[52] Flock T (2015). Universal allosteric mechanism for Gα activation by GPCRs. Nature.

[53] Maeda S (2019). Structures of the M1 and M2 muscarinic acetylcholine receptor/G-protein complexes. Science.

[54] Cotecchia S (2015). The alpha1-adrenergic receptors in cardiac hypertrophy: Signaling mechanisms and functional implications. Cell. Signal..

[55] Perez-Aso M (2013). The three α1-adrenoceptor subtypes show different spatio-temporal mechanisms of internalization and ERK1/2 phosphorylation. Biochim. Biophys. Acta (BBA) - Mol. Cell Res..

[56] Carpenter B, Tate CG (2017). Expression and purification of mini G proteins from Escherichia coli. Bio-protocol.

[57] Lei J, Frank J (2005). Automated acquisition of cryo-electron micrographs for single particle reconstruction on an FEI Tecnai electron microscope. J. Struct. Biol..

[58] Zheng SQ (2017). MotionCor2: anisotropic correction of beam-induced motion for improved cryo-electron microscopy. Nat Methods.

[59] Zhang K (2016). Gctf: Real-time CTF determination and correction. J. Struct. Biol..

[60] Kooistra AJ (2021). GPCRdb in 2021: integrating GPCR sequence, structure and function. Nucleic Acids Res..

[61] Tunyasuvunakool, K. et al. Highly accurate protein structure prediction for the human proteome. *Nature*. **596**, 590–596 (2021). 10.1038/s41586-021-03828-1 PMC8387240 34293799

[62] Waterhouse A (2018). SWISS-MODEL: homology modelling of protein structures and complexes. Nucleic Acids Res..

[63] Pettersen EF (2004). UCSF Chimera-a visualization system for exploratory research and analysis. J. Comput. Chem..

[64] Afonine PV (2018). Real-space refinement in PHENIX for cryo-EM and crystallography. Acta Crystallogr. D Struct. Biol..

[65] Emsley P, Cowtan K (2004). Coot: model-building tools for molecular graphics. Acta Crystallogr. D Biol. Crystallogr..

