# Supplementary material for: Structural basis of α1A-adrenergic receptor activation and recognition by an extracellular nanobody
Source: Nat Commun. 2023 Jun 20;14:3655. doi: 10.1038/s41467-023-39310-x (PMC10282093; doi:10.1038/s41467-023-39310-x)
Supplement: Supplementary file 1 — Supplementary Information [file 41467_2023_39310_MOESM1_ESM.pdf]

Supplementary Information

Structural basis of α_{1A}-adrenergic receptor activation and recognition by an extracellular nanobody

Yosuke Toyoda, Angqi Zhu, Fang Kong, Sisi Shan, Jiawei Zhao, Nan Wang, Xiaoou Sun, Linqi Zhang, Chuangye Yan, Brian K. Kobilka, Xiangyu Liu

This PDF file includes:
Supplementary Figures 1-10
Supplementary Tables 1-2

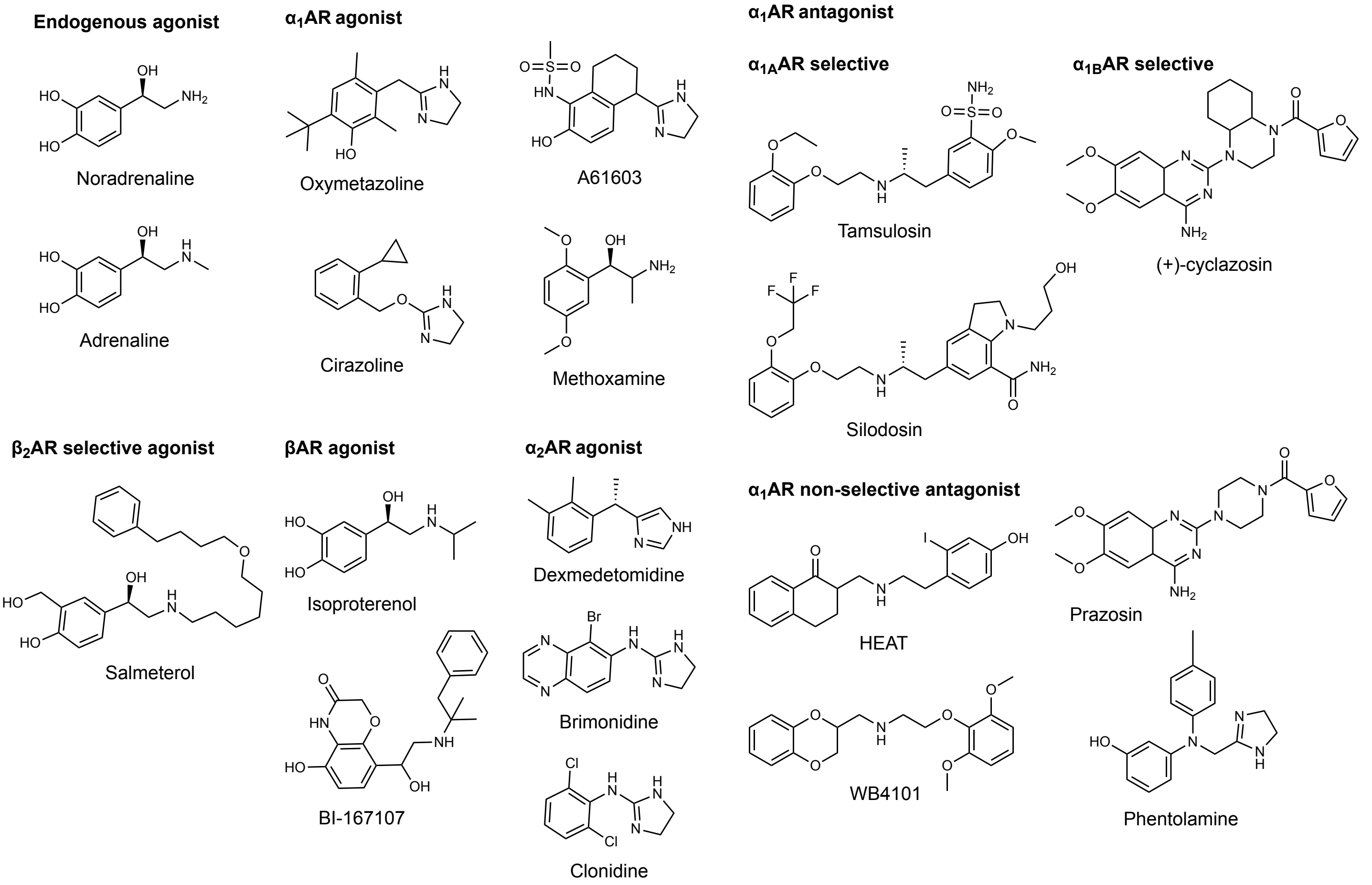

Supplementary Figure 1: Chemical structures of the representative adrenergic receptor agonists and antagonists.

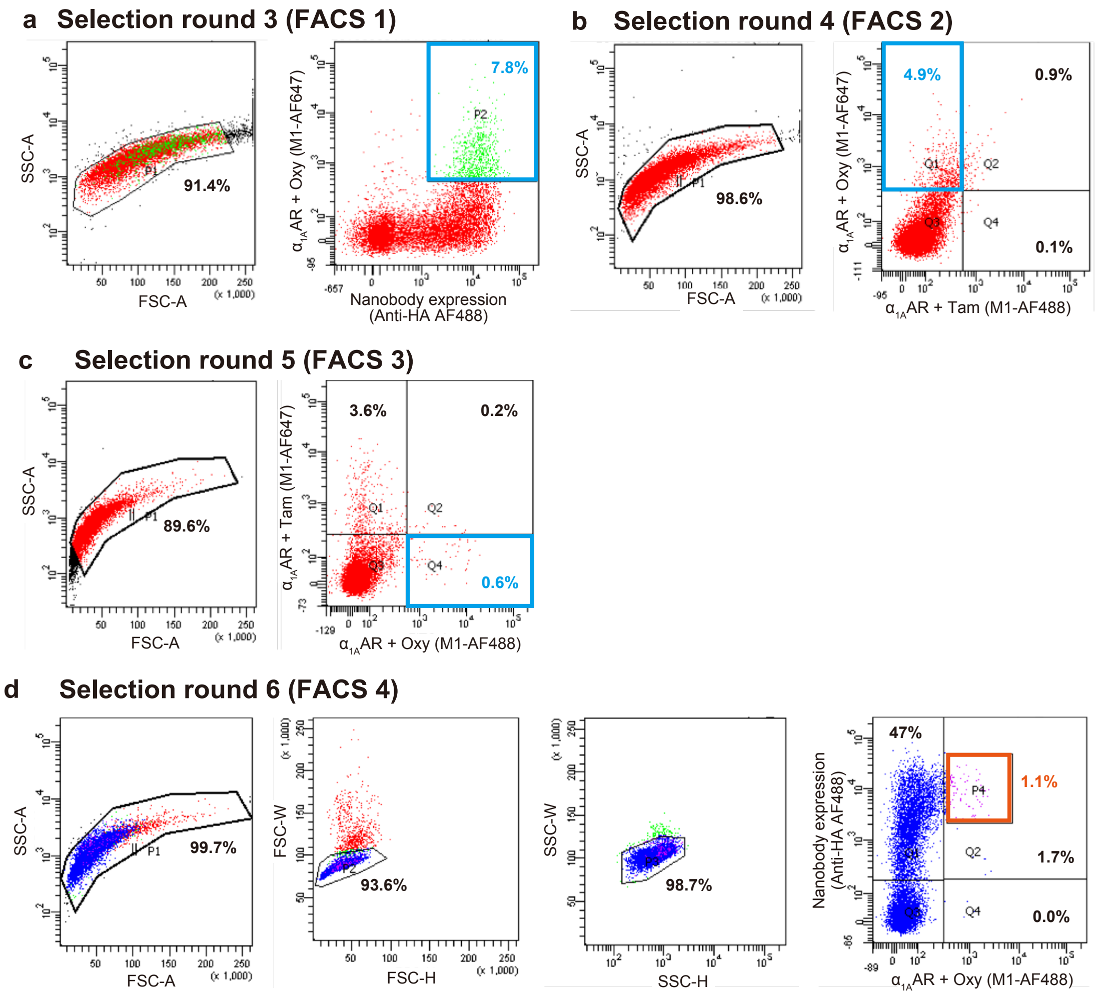

Supplementary Figure 2: Flow cytometry for selection of Nb29.

Gating scheme for fluorescence-activated cell sorting (FACS) with oxymetazoline (oxy)- and tamsulosin (tam)-bound α_{1A}AR. **a**: Selection round 3 (FACS1), **b**: Selection round 4 (FACS2), **c**: Selection round 5 (FACS3) and **d**: Selection round 6 (FACS4). Sorted yeast cells were indicated by surrounding in cyan squares for selection rounds 3-5 or red squares for selection round 6.

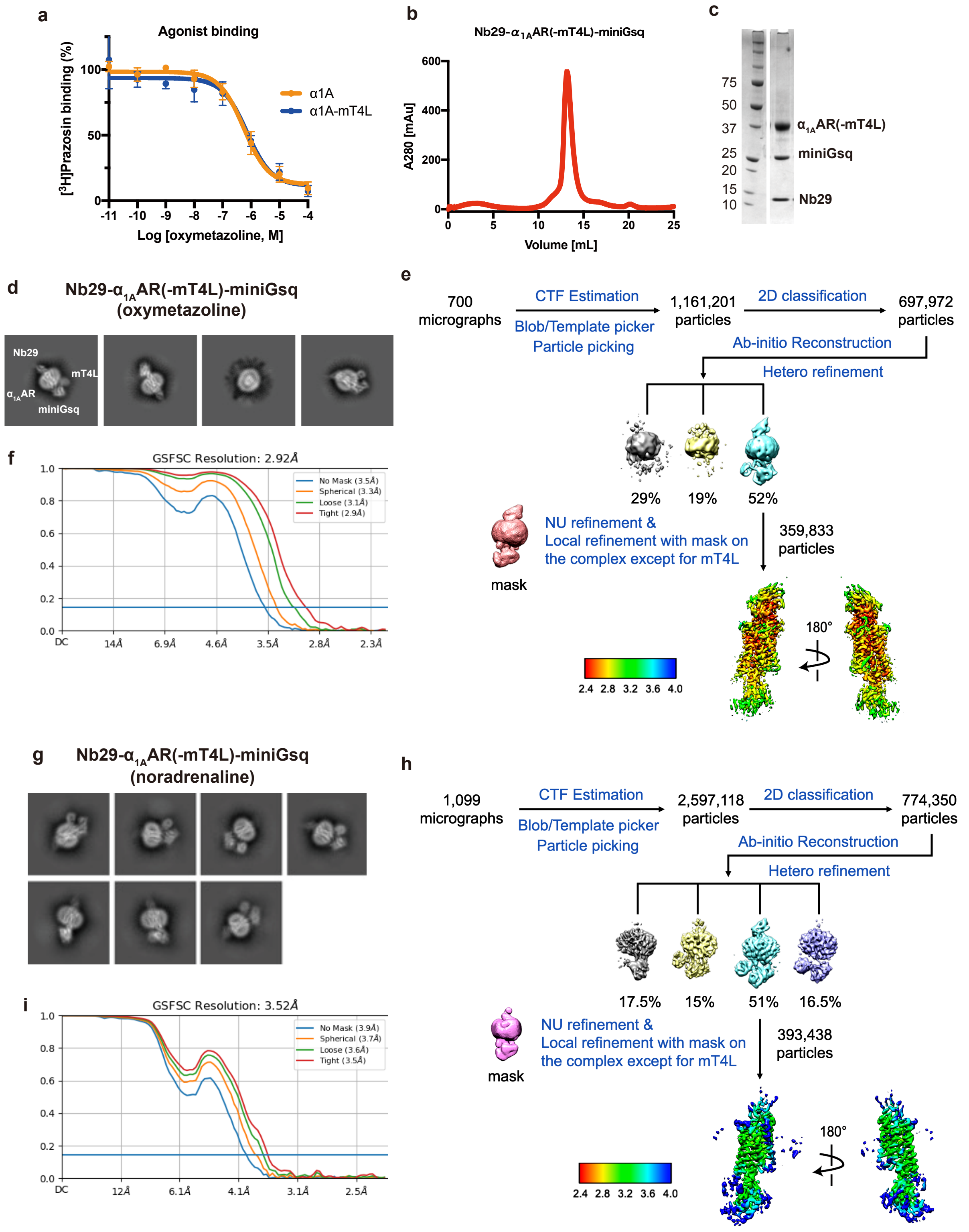

Supplementary Figure 3: Purification, cryo-EM data processing of Nb29-α$_1$AR-miniGsq complex.

a, [^{3}H]prazosin radioligand competition binding for oxymetazoline of α$_{1A}$AR and α$_{1A}$AR-mT4L in *Sf9* membrane against oxymetazoline. The data represent mean ± s.e.m. of n = 3 independent measurements. K_i values are 511 ± 1.2 nM (α$_{1A}$AR) and 647 ± 1.4 nM (α$_{1A}$AR-mT4L). **b**, Diagram of size-exclusion chromatography of Nb29-α$_{1A}$AR-mT4L-miniGsq (red line). **c**, Coomassie blue–stained SDS-PAGE gel of Nb29-α$_{1A}$AR-mT4L-miniGsq. The raw image of the gel is provided in the Source Data file. **d, g**, Representative 2D classifications from different views. **e, h**, Workflow of cryo-EM data processing and local resolution maps. **f**, **i**, Gold standard FSC curve indicates the overall nominal resolution. Oxymetazoline-bound (**d, e** and **f**) and noradrenaline-bound states (**g**, **h** and **i**) are shown. Source data are provided in the Source Data file.

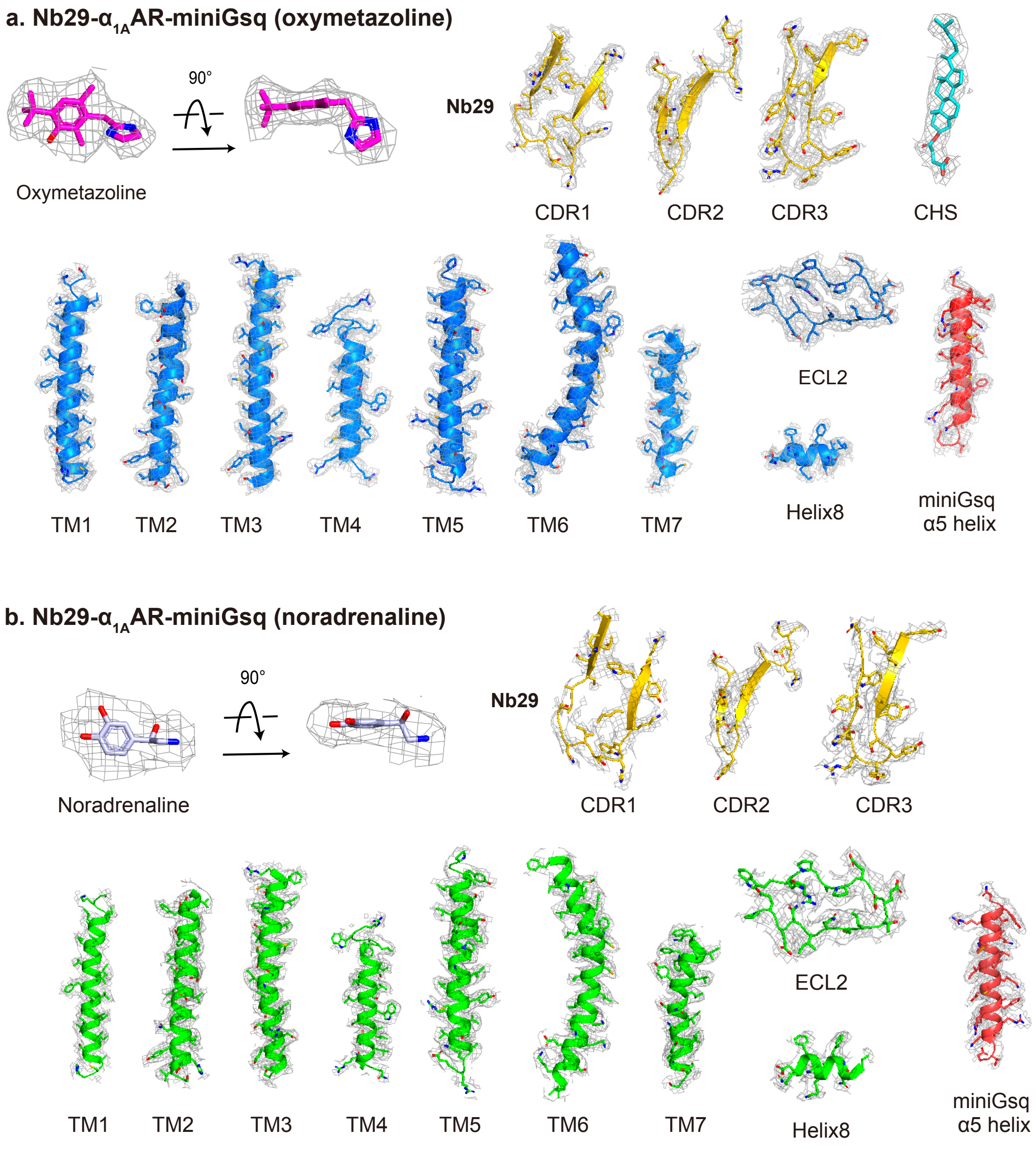

Supplementary Figure 4: Cyo-EM map quality and refined structures of Nb29-α$_1$AR-mT4L-miniGsq complex.
Cryo-EM density map and the model of Nb29-α$_{1A}$AR- miniGsq are shown for ligands, CDRs of Nb29, all transmembrane helices, ECL2 and helix 8 of α$_1$AR, α5 helix of miniGsq and CHS (**a**: oxymetazoline-bound and **b**: noradrenaline-bound states). The density map is shown in gray mesh, contoured at 1.5σ.

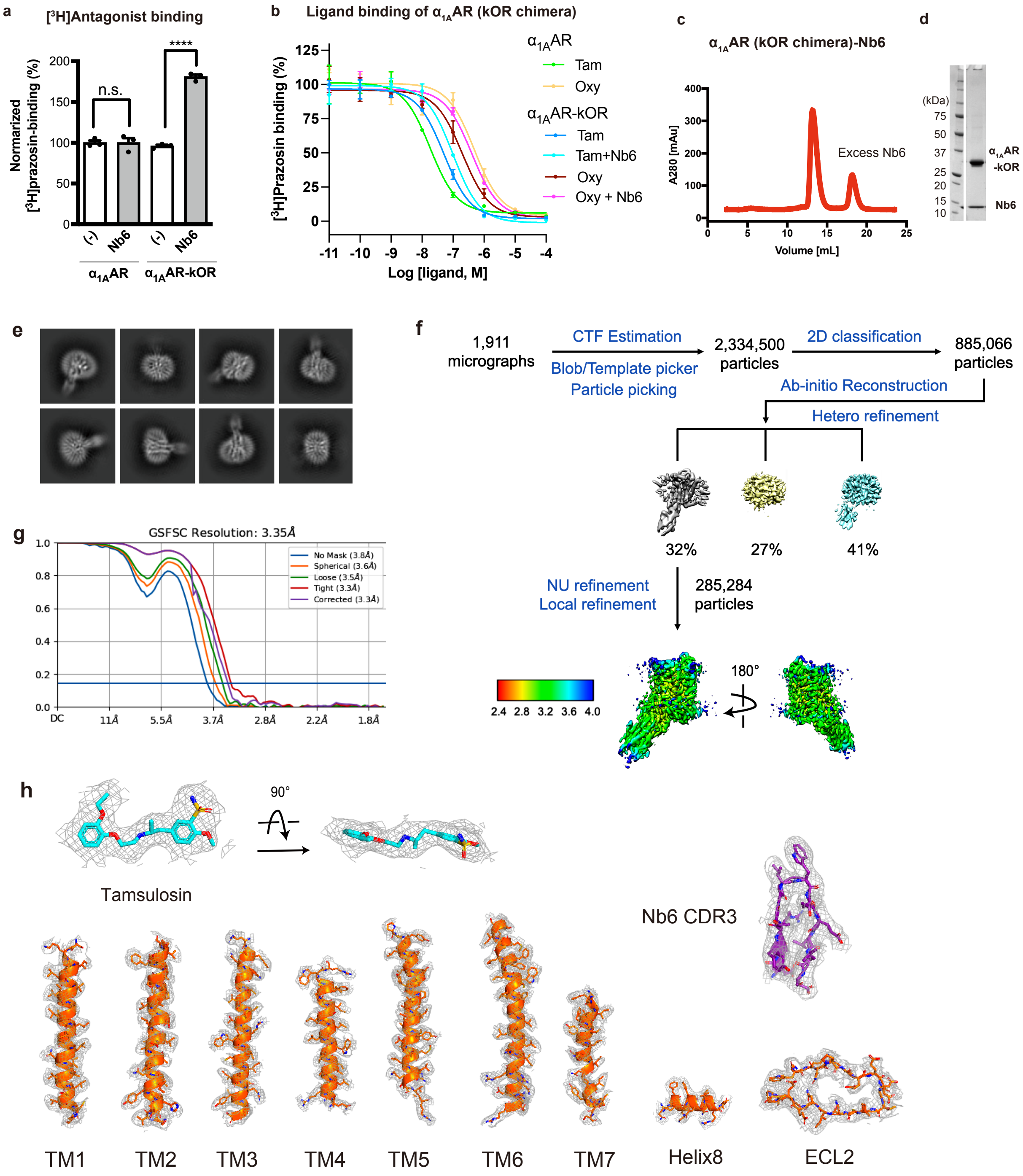

Supplementary Figure 5: Purification and cryo-EM data processing of α_1AR-Nb6 complex.
a, [^{3}H]prazosin binding of α_{1A}AR and α_{1A}AR-Nb6 mutants in *Sf9* membrane with or without 5 μM Nb6. ****, *P* value = < 0.0001 by two-tailed unpaired *t*-test. n.s., not significant. The data represent mean ± s.e.m. of *n* = 3 independent measurements. **b**, [^{3}H]prazosin radioligand competition binding for oxymetazoline and tamsulosin of α_{1A}AR (control) and α_{1A}AR-Nb6 in *Sf9* membrane. 5 μM concentration of Nb6 was used for in the presence of Nb6. The data represent mean ± s.e.m. of three independent measurements. K_i values of α_{1A}AR are 17 ± 1.3 nM (Tam) and 442 ± 1.4 nM (Oxy). K_i values of α_{1A}AR-kOR are 46 ± 1.1 nM (Tam), 91 ± 1.2 nM (Tam+Nb6), 183 ± 1.3 nM (oxy) and 365 ± 1.2 nM (oxy+Nb6). **c**, Diagram of size-exclusion chromatography of α_1AR-Nb6 (red line). **d**, Coomassie blue-stained SDS-PAGE gel of α_1AR-Nb6 complex. The raw image of the gel is provided in the Source Data file. **e,** Representative 2D classifications from different views. **f,** Workflow of cryo-EM data processing and local resolution maps. **g**, Gold standard FSC curve indicates the overall nominal resolution. **h**, Cryo-EM density map and the model of α_1AR-Nb6 are shown for ligands, CDR3 of Nb6, all transmembrane helices, ECL2 and helix 8 of α_1AR. The density map is shown in gray mesh, contoured at 1.5σ. Source data are provided in the Source Data file.

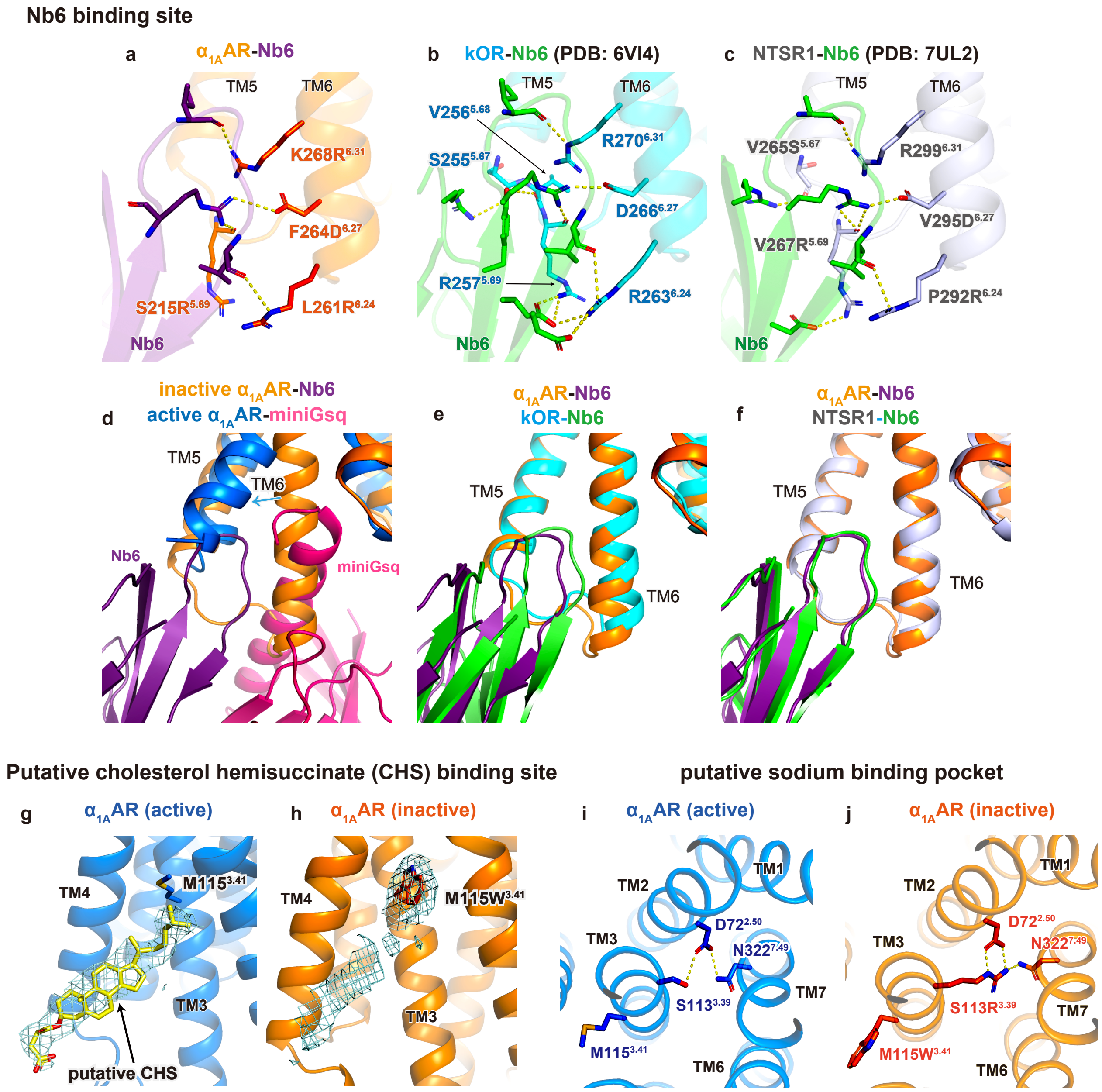

Supplementary Figure 6: Nb6 binding interaction, putative sodium-binding pocket and CHS binding site. **a-c,** Interactions of Nb6 for α_{1A}AR-Nb6, kOR-Nb6 (PDB ID: 6VI4) and NTSR1-Nb6 (PDB ID: 7UL2) complexes. Residues are shown in stick representation. Polar interactions are shown as yellow dash lines. **d-f,** Structural comparisons of α_{1A}AR-Nb6 and Nb29-α_{1A}AR-miniGsq complexes (**d**); α_{1A}AR-Nb6 and kOR-Nb6 complexes (**e**); α_{1A}AR-Nb6 and NTSR1-Nb6 complexes (**f**). **g-h,** Comparisons of cholesterol hemisuccinate (CHS) binding site for oxymetazoline-bound active states (**g**) and tamsulosin-bound inactive α_{1A}AR (**h**). Densities are shown as cyan mesh, contoured at 1.5σ. **i-j,** Comparisons of sodium binding sites for active and inactive α_{1A}ARs. Polar interactions are shown as yellow dash lines.

Ligand binding

Nb29										H.B.			S.B.										S.B.	cat-π		
Tam	V.W.	V.W.	V.W.	V.W.		H.B.	V.W.		H.B.							V.W.			V.W.	V.W.	V.W.			V.W.		V.W.
Oxy						H.B.	V.W.	H.B.				V.W.		V.W.	V.W.	V.W.		V.W.	V.W.	V.W.	V.W.				π-π	V.W.
NA						H.B.		V.W.								H.B.		V.W.	V.W.	V.W.	V.W.				cat-π, H.B.	V.W.
B/W	2.61	2.64	2.65	3.28	3.29	3.32	3.33	3.36	45.50	45.51	45.52	ECL 2	ECL 2	5.38	5.39	5.42	5.43	6.48	6.51	6.52	6.55	6.58	7.32	7.35	7.39	7.43
α1A	S 83	F 86	E 87	W 102	A 103	D 106	V 107	C 110	C 176	Q 177	I 178	N 179	E 180	Y 184	V 185	S 188	A 189	W 285	F 288	F 289	M 292	G 295	E 305	F 308	F 312	Y 316
α1B	S	L	E	W	A	D	V	C	C	G	V	T	E	Y	A	S	S	W	F	F	L	G	D	F	F	Y
α1D	S	M	E	W	A	D	V	C	C	G	I	T	E	Y	A	S	S	W	F	F	L	G	E	F	F	Y
α2A	S	N	E	Y	L	D	V	C	C	E	I	N	D	Y	V	S	C	W	F	F	Y	T	R	F	F	Y
α2B	S	N	E	Y	L	D	V	C	C	K	L	N	Q	Y	I	S	S	W	F	F	Y	G	H	F	F	Y
α2C	S	N	E	Y	L	D	V	C	C	G	L	N	D	Y	I	S	C	W	F	F	Y	Y	G	F	F	Y
β1	G	I	V	W	T	D	V	V	C	D	F	V	T	Y	A	S	S	W	F	F	N	K	D	F	N	Y
β2	G	H	I	W	T	D	V	V	C	D	F	F	T	Y	A	S	S	W	F	F	N	H	K	Y	N	Y
β3	A	L	A	W	T	D	V	V	C	A	F	A	S	Y	V	S	S	W	F	F	N	R	G	F	N	Y

Supplementary Figure 7: Sequence alignment of human adrenergic receptor subtypes and ligand-binding pockets of α_1AR. Amino acid sequence alignment of nine human adrenergic receptor subtypes involved in ligand binding for noradrenaline (NA), oxymetazoline (oxy) and tamsulosin (Tam). Conserved amino acids with α_1AR at the same B/W position were shown as a red character. B/W indicates Ballesteros-Weinstein numbering for GPCRs from GPCRdb (gpcrdb.org) [59]. H.B., V.W., π-π, cat-π, and S.B. indicate interactions of hydrogen bond, van der Waals, π-π stacking, cation-π interaction, and salt-bridge, respectively.

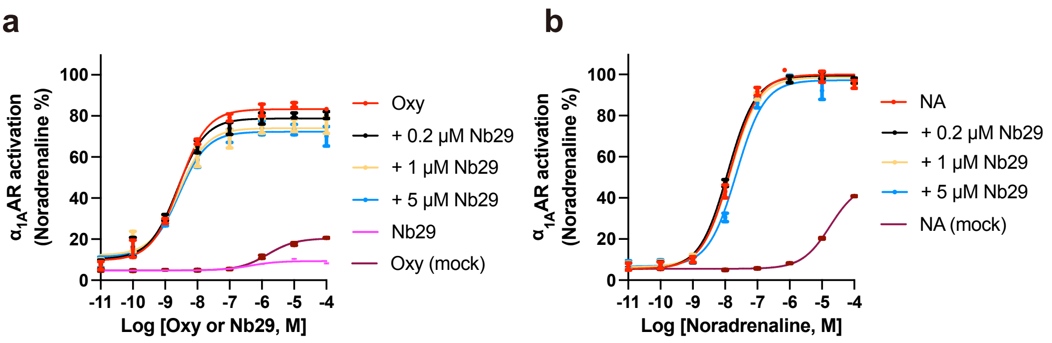

c

Ligand (+ Nb29)	pEC_{50}	E_{max} (% NA)
Oxy	8.56 ± 0.063	83.3 ± 1.1
+ 0.2 μM	8.62 ± 0.082	78.8 ± 1.4
+ 1 μM	8.57 ± 0.13	74.0 ± 1.9
+ 5 μM	8.57 ± 0.11	72.3 ± 1.7
NA	7.85 ± 0.045	100.0 ± 1.2
+ 0.2 μM	7.91 ± 0.033	99.5 ± 0.89
+ 1 μM	7.84 ± 0.030	98.5 ± 0.80
+ 5 μM	7.65 ± 0.057	97.2 ± 1.5

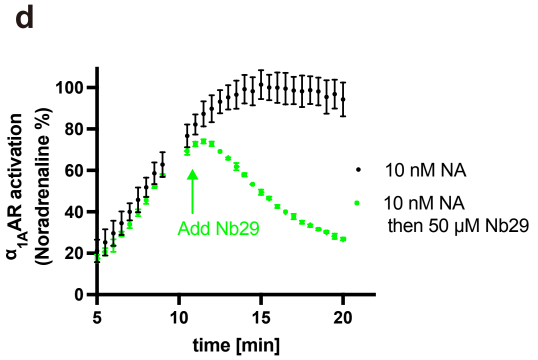

Supplementary Figure 8: Functional analyses of Nb29.

a, **b,** Signaling activities of α_{1A}AR upon oxymetazoline (Oxy, **a**) and noradrenaline (NA, **b**) stimulation were evaluated by Glo-sensor cAMP assay with the engineered Gsq protein in HEK293T cells. Nb29 added at the same time, The data represent mean ± s.e.m. of *n* = 3 independent measurements in duplicate. The mock was used as a negative control (*n* = 3 independent measurements). **c,** Summary of signaling assay of **a** and **b**. **d,** Time-course of signaling activity of α_{1A}AR upon noradrenaline (NA) stimulation. The green plot indicates that Nb29 (50 μM) was added after 10 min stimulation by noradrenaline (10 μM). The data represent mean ± s.e.m. of three independent measurements. Source data are provided in the Source Data file.

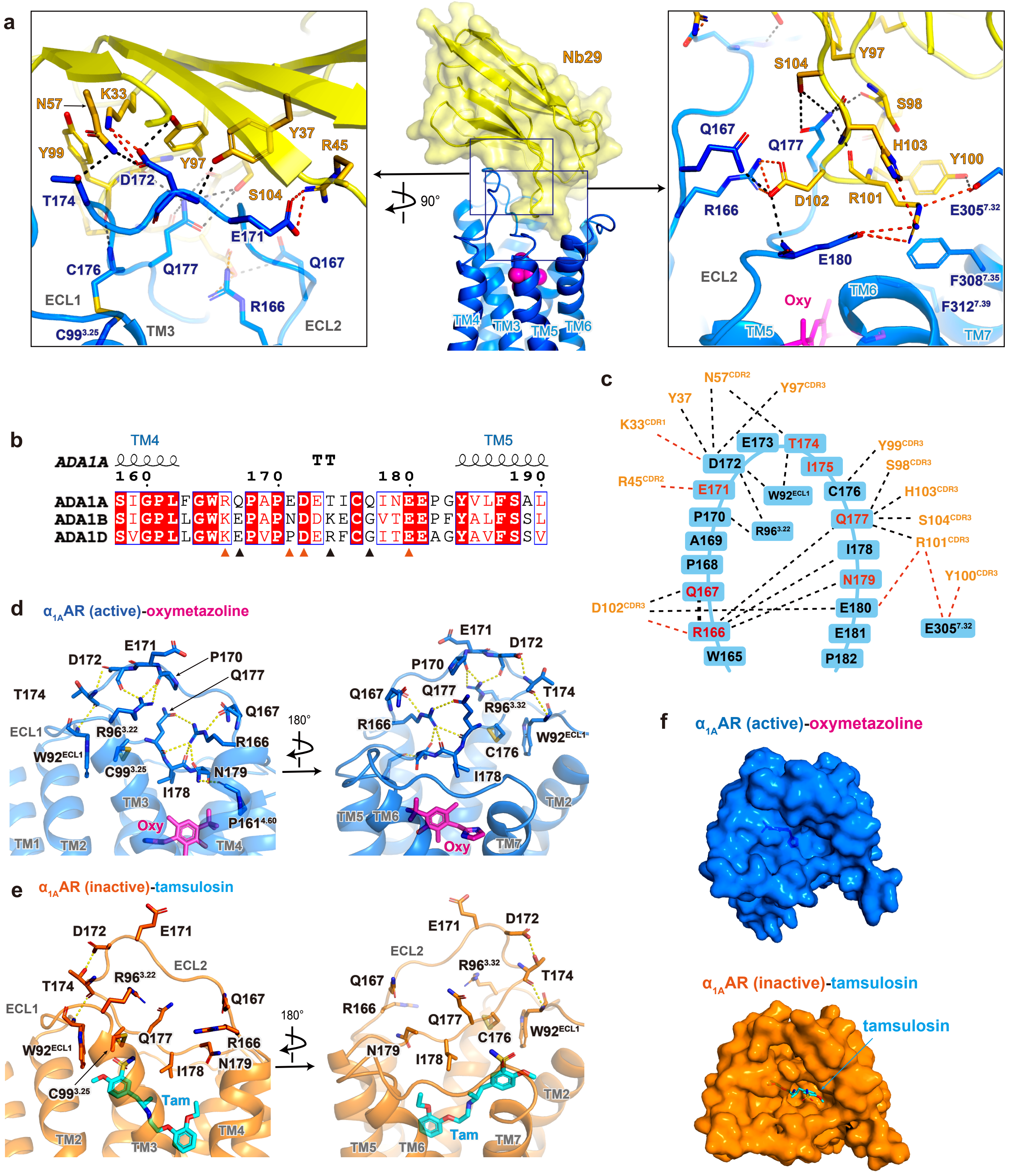

Supplementary Figure 9: Nb29 binding interaction and conformational change of ECL2.

a, Interaction of Nb29 and α_{1A}AR are shown in stick representation. Hydrogen bonds and salt bridges are shown as black and orange dash lines, respectively. **b**, Sequence alignment of ECLs for α_1AR subtypes. Amino acids involved in hydrogen bonds and salt bridges between α_1AR and Nb29 are marked with black and orange triangles, respectively. **c,** Diagram of the interaction of Nb29 and α_{1A}AR. Hydrogen bonds and salt bridges are shown as black and orange dash lines, respectively. The red character indicates unique residues to α_{1A}AR. **d, e,** Polar interaction networks in ECLs for active and inactive α_{1A}ARs. Polar interactions are shown as yellow dash lines. **f,** Surface representations of top views of active α_{1A}AR (blue) bound to oxymetazoline (magenta) and inactive α_{1A}AR (orange) tamsulosin (cyan).

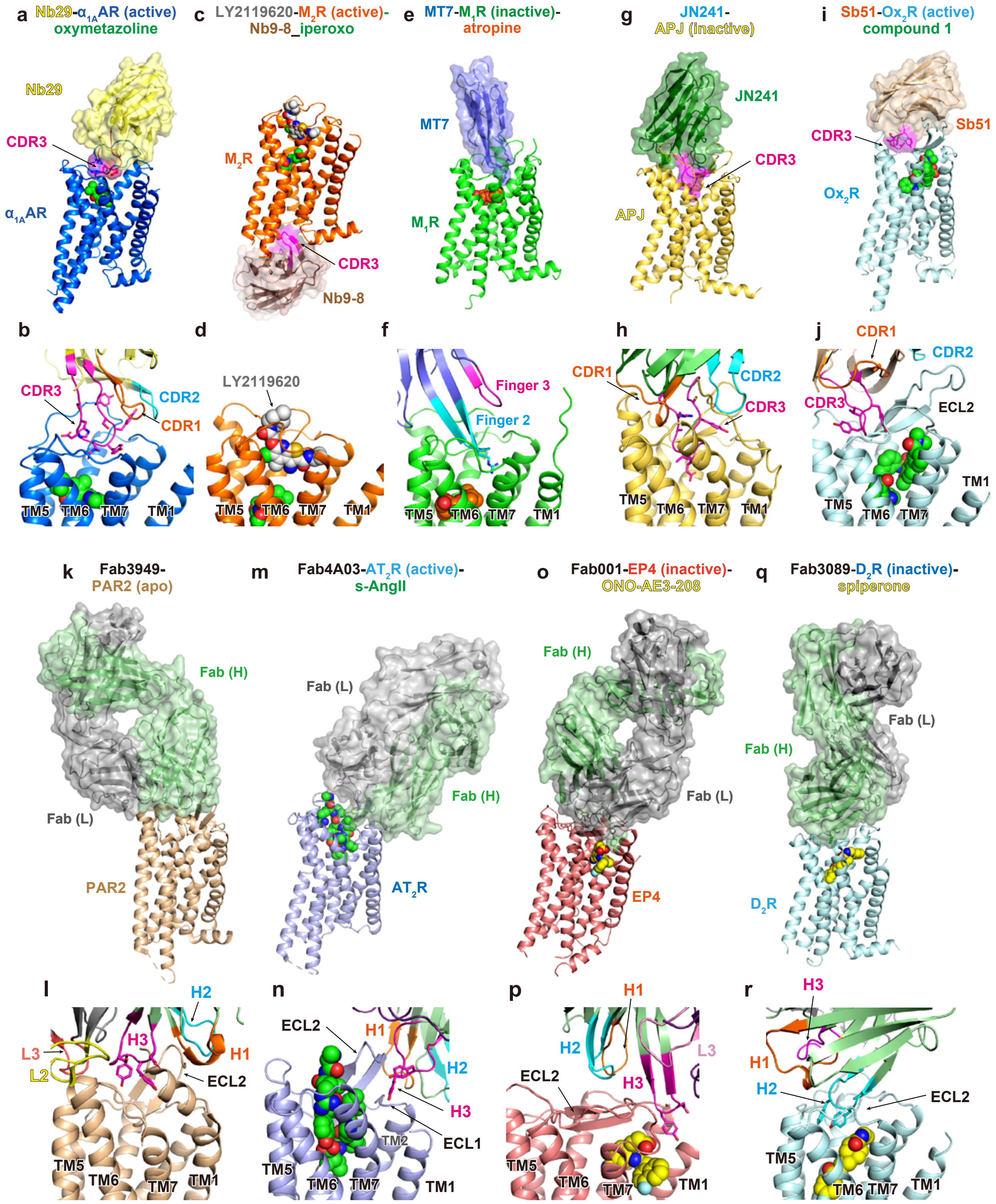

Supplementary Figure 10: Binding modes of class A GPCRs in complex with the extracellular binders.

Overall structures and close-ups of GPCRs in complex with nanobodies are shown as cartoon representations. **a, b**, α_1AR in complex with Nb29. **c, d,** muscarinic acetylcholine receptor M2 (M_2R) bound to positive allosteric modulator and Nb9-8 (PDB ID: 4MQT). **e, f,** muscarinic acetylcholine receptor M1 (M_1R) bound to muscarinic toxin MT7 and atropine (PDB ID: 6WJC). **g, h,** apelin receptor APJ in complex with antagonistic Nb JN241 (PDB ID: 6KNM). **i, j,** orexin receptor 2 in complex with Sb51 (Ox_2R, PDB ID: 7L1V). **k, l,** protease-activated receptor Par2 in complex with inhibitory Fab3969 (PDB ID: 5NJ6). **m, n,** angiotensin receptor type 2 AT2 in complex with Fab4A03 (PDB ID: 5XJM). **o, p,** prostaglandin E receptor EP4 in complex with inhibitory Fab001 (PDB ID: 5YWY). **q, r,** dopamine receptor D2 in complex with Fab3085 (PDB ID: 7DFP).

G protein binding

α_{1A}AR-mGsq	✔	✔				✔			✔	✔		✔	✔
α_{2A}AR-Go		✔											
β_2AR-Gs	✔		✔	✔	✔		✔	✔	✔				
B/W	3.50	3.53	3.54	3.55	5.64	5.67	5.68	5.71	6.36	7.55	7.56	8.47	8.48
α1A	R124	G127	V128	S129	V210	R213	E214	G217	T273	C328	S329	S330	Q331
α1B	R	G	V	R	V	R	T	N	T	C	S	S	K
α1D	R	G	V	R	V	S	T	S	T	C	S	S	R
α2A	R	S	I	T	I	R	R	V	V	I	F	N	H
α2B	R	A	V	S	I	R	S	R	V	V	F	N	Q
α2C	R	S	V	T	V	L	R	T	V	V	F	N	Q
β1	R	A	I	T	E	K	Q	K	T	R	-	S	P
β2	R	A	I	T	E	R	Q	K	T	R	-	S	P
β3	R	A	V	T	V	R	Q	L	T	R	-	S	P

Supplementary Figure 11: Sequence alignment of human adrenergic receptor subtypes involved in G protein coupling. Amino acid sequence alignment of nine human adrenergic receptor subtypes involved in G protein coupling interfaces. Check marks indicate polar interactions. B/W indicates Ballesteros-Weinstein numbering for GPCRs from GPCRdb (gpcrdb.org).

Supplementary Table 1. Functional characterization of Nb29 against α_1ARs

a, On-yeast titration of Nb29 for α_{1A}AR.

	K_d values [nM]	Bmax [%]	*n*
Apo	209 ± 288	35.8 ± 25.7	3
Oxymetazoline	37.6 ± 25.7	75.5 ± 13.8	3
Noradrenaline	38.8 ± 38.5	10.9 ± 3.4	3
Tamsulosin	16.7 ± 12.9	8.90 ± 1.8	3
Phentolamine	296.2 ± 666	24.8 ± 4.0	3

b, ^{3}H-prazosin radioligand competition binding of α_1AR subtype for oxymetazoline in *Sf9* membranes.

	K_i	pK_i	*n*
α_{1A}AR	650 nM	6.19 ± 0.16	3
α_{1A}AR + Nb29	156 nM	6.81 ± 0.091	3
α_{1B}AR	6.6 μM	5.18 ± 0.35	3
α_{1B}AR + Nb29	3.2 μM	5.50 ± 0.21	3
α_{1D}AR	26 μM	4.59 ± 0.22	3
α_{1D}AR + Nb29	60 μM	4.22 ± 0.36	3
Nb29 for α_{1A}AR	41 μM	4.39 ± 0.40	3

c, ^{3}H-prazosin radioligand competition binding of the purified α_{1A}AR-bound anti-Flag M1 affinity resin for oxymetazoline (Oxy), noradrenaline (NA) or Nb29.

	K_i	pK_i	*n*
Oxymetazoline (Oxy)	1.3 μM	5.90 ± 0.19	6
Oxy + 5 μM Nb29	38 nM	7.42 ± 0.14	6
Oxy + 20 μM Nb29	109 nM	6.96 ± 0.37	6
Nb29	6.4 μM	5.50 ± 0.21	6
Noradrenaline (NA)	6.0 μM	5.22 ± 0.15	6
NA + 5 μM Nb29	2.6 μM	5.58 ± 0.18	3
NA + 20 μM Nb29	10 μM	4.99 ± 0.23	6

a, On-yeast titration to estimate the affinity of Nb29 for α_{1A}AR, evaluated by flow cytometry. The ratio of Nb29-displayed yeast cells bound purified α_{1A}AR in the presence or absence of 500 μM ligands was analyzed. The data represent mean ± s.e.m. of three independent measurements. **b**, ^{3}H-prazosin radioligand competition binding of α_1AR subtype for oxymetazoline in *Sf9* membranes. Samples in the presence of Nb29 were used at 5 μM concentration of Nb29. The data represent mean ± s.e.m. of three independent measurements. **c,** ^{3}H-prazosin radioligand competition binding of the purified α_{1A}AR-bound M1-Flag affinity resin for oxymetazoline (Oxy), noradrenaline (NA) or Nb29. The data represent mean ± s.e.m.

Supplementary Table 2. Cryo-EM data collection, refinement and validation statistics

	Nb29-α_{1A}AR-miniGsq bound to oxymetazoline (EMDB-33924) (PDB 7YM8)	Nb29-α_{1A}AR-miniGsq bound to noradrenaline (EMDB-33928) (PDB 7YMH)	α_{1A}AR-Nb6 bound to tamsulosin (EMDB-33930) (PDB 7YMJ)
**Data collection and processing**			
Microscope	Titan Krios G3i	Titan Krios G3	Titan Krios G4
Detector	Gatan K3	Gatan K3	Falcon-4
Magnification	81,000	64,000	96,000
Voltage (kV)	300	300	300
Electron exposure (e–/Å^2)	50	50	50
Defocus range (μm)	-1.3~-1.8	-1.3~-1.8	-1.3~-1.8
Pixel size (Å)	1.083	1.098	0.860
Symmetry imposed	C1	C1	C1
Initial particle images (no.)	1,161 k	2,597 k	2,334 k
Final particle images (no.)	359 k	393 k	285 k
Map resolution (Å)	2.92	3.52	3.35
FSC threshold	0.143	0.143	0.143
Map resolution range (Å)	50~2.4	50~2.9	50~2.7
**Refinement and validation**			
Initial model used (PDB code)	α_{1A}AR: active α_{1A}AR homology model (gpcrdb.org) Nb29: homology model (Swiss model) miniGsq: 5G53	This study (PDB: 7YM8)	α_{1A}AR: α_{1A}AR-kOR homology model (AlphaFold2) Nb6: 6VI4
Map sharpening *B* factor (Å^2)	-131.6	-152.2	-195.5
Model composition			
Non-hydrogen atoms	4288	3948	3085
Protein residues	556	542	396
Ligands	Oxymetazoline	Noradrenaline	Tamsulosin
B factors (Å^2)			
Protein	33.64	86.68	59.55
Ligand	32.12	42.17	33.41
R.m.s. deviations			
Bond lengths (Å)	0.005	0.004	0.005
Bond angles (°)	0.836	0.768	0.870
Validation			
MolProbity score	1.48	1.48	1.09
Clashscore	4.03	3.56	1.30
Poor rotamers (%)	0.00	0.00	0.00
Ramachandran plot			
Favored (%)	95.77	95.23	96.41
Allowed (%)	4.23	4.77	3.59
Disallowed (%)	0.00	0.00	0.00
