# Supplementary material for: Structural basis of α1A-adrenergic receptor activation and recognition by an extracellular nanobody
Source: Nat Commun. 2023 Jun 20;14:3655. doi: 10.1038/s41467-023-39310-x (PMC10282093; doi:10.1038/s41467-023-39310-x)
Supplement: Supplementary file 3 — Reporting Summary [file 41467_2023_39310_MOESM3_ESM.pdf]

nature portfolio

Corresponding author(s): Yosuke Toyoda, Chuangye Yan, Brian K. Kobilka, Liu Xiangyu.

Last updated by author(s): May 22, 2023

Reporting Summary

Nature Portfolio wishes to improve the reproducibility of the work that we publish. This form provides structure for consistency and transparency in reporting. For further information on Nature Portfolio policies, see our Editorial Policies and the Editorial Policy Checklist.

Statistics

For all statistical analyses, confirm that the following items are present in the figure legend, table legend, main text, or Methods section.

n/a	Confirmed	
☐	☒	The exact sample size (*n*) for each experimental group/condition, given as a discrete number and unit of measurement
☐	☒	A statement on whether measurements were taken from distinct samples or whether the same sample was measured repeatedly
☐	☒	The statistical test(s) used AND whether they are one- or two-sided<br>*Only common tests should be described solely by name; describe more complex techniques in the Methods section.*
☒	☐	A description of all covariates tested
☒	☐	A description of any assumptions or corrections, such as tests of normality and adjustment for multiple comparisons
☐	☒	A full description of the statistical parameters including central tendency (e.g. means) or other basic estimates (e.g. regression coefficient) AND variation (e.g. standard deviation) or associated estimates of uncertainty (e.g. confidence intervals)
☐	☒	For null hypothesis testing, the test statistic (e.g. F, t, r) with confidence intervals, effect sizes, degrees of freedom and P value noted<br>*Give P values as exact values whenever suitable.*
☒	☐	For Bayesian analysis, information on the choice of priors and Markov chain Monte Carlo settings
☒	☐	For hierarchical and complex designs, identification of the appropriate level for tests and full reporting of outcomes
☒	☐	Estimates of effect sizes (e.g. Cohen's *d*, Pearson's *r*), indicating how they were calculated

Our web collection on statistics for biologists contains articles on many of the points above.

Software and code

Policy information about availability of computer code

Data collection	AutoEMation V2
Data analysis	GraphPad Prizm v9, FlowJo v10, MortionCor2 v1, cryoSPARC v3.2, COOT v0.9.6, PHENIX v1.18, USCF ChimeraX v1.3, AlphoFold v2, PyMOL v2.4.0

For manuscripts utilizing custom algorithms or software that are central to the research but not yet described in published literature, software must be made available to editors and reviewers. We strongly encourage code deposition in a community repository (e.g. GitHub). See the Nature Portfolio guidelines for submitting code & software for further information.

Data

Policy information about availability of data

All manuscripts must include a data availability statement. This statement should provide the following information, where applicable:

- Accession codes, unique identifiers, or web links for publicly available datasets
- A description of any restrictions on data availability
- For clinical datasets or third party data, please ensure that the statement adheres to our policy

The cryo-EM density maps for the Nb29-α1AAR-miniGsq complexes bound to oxymetazoline and noradrenaline, and α1AAR-Nb6 complex have been deposited in the Electron Microscopy Data Bank under accession codes EMDB-33924, EMD-33928 and EMD-33930, respectively. The coodinates for the models have been deposited in the Protein Data Bank under accession coded 7YM8, 7YMH and 7YMJ, respectively.

Previously published structures can be accessed via accession codes: 5G53, 6VI4, 7EJ0, 7BU6, 7EJK, 7B6W, 2RH1, 4MQT, 3UON, 6WJC, 5CXV, 6MXT, 7EJ0, 7UL2, 6WJC, 6KNM, 7L1V, 5YWY, 7DFP

Human research participants

Policy information about studies involving human research participants and Sex and Gender in Research.

Reporting on sex and gender	No human research was performed in this study.
Population characteristics	This study did not involve human research participants.
Recruitment	No human subjects in this study.
Ethics oversight	No human subjects in this study.

Note that full information on the approval of the study protocol must also be provided in the manuscript.

Field-specific reporting

Please select the one below that is the best fit for your research. If you are not sure, read the appropriate sections before making your selection.

☒ Life sciences ☐ Behavioural & social sciences ☐ Ecological, evolutionary & environmental sciences

For a reference copy of the document with all sections, see nature.com/documents/nr-reporting-summary-flat.pdf

Life sciences study design

All studies must disclose on these points even when the disclosure is negative.

Sample size	A sample size of n=3 is commonly used in biological studies. All experiments were performed at least three independent biological experiment. The sample particles were automatically picked from the collected cryo-EM micrographs by software packages.
Data exclusions	No data were excluded from the analyses.
Replication	All experiments were repeated for at least three independent experiments and successfully reproduced.
Randomization	As single particle analysis is automatically processed by software packages, randomization is not relevant to this study.
Blinding	Since no subjective assessment was required, blinding was not required.

Reporting for specific materials, systems and methods

We require information from authors about some types of materials, experimental systems and methods used in many studies. Here, indicate whether each material, system or method listed is relevant to your study. If you are not sure if a list item applies to your research, read the appropriate section before selecting a response.

Materials & experimental systems

n/a	Involved in the study
☐	☒ Antibodies
☐	☒ Eukaryotic cell lines
☒	☐ Palaeontology and archaeology
☒	☐ Animals and other organisms
☒	☐ Clinical data
☒	☐ Dual use research of concern

Methods

n/a	Involved in the study
☒	☐ ChIP-seq
☐	☒ Flow cytometry
☒	☐ MRI-based neuroimaging

Antibodies

Antibodies used	Anti-FLAG M1 antibodies (Purified from mouse monoclonal IgG2a M1 Hybridoma obtained from Dr. Brian Kobilka at Stanford, PMID 17962520), Anti-FLAG-488 or -647 M1 antibody (Anti-FLAG M1 antibodies were labeled with Alexa Fluor-488 or -647 NHS ester), Alexa Fluor 488 conjugated anti-HA mAb (Cell Signaling Tech., 2350S), Alexa Fluor 647 conjugated anti-HA mAb (Cell Signaling Tech., 3444S).

March 2021

	Yeast surface displayed synthetic nanobody library was obtained from Drs. Andrew Kruse and Aashish Manglik (MacMahon et al. Nat Struct Mol Biol, 2018. p289-296).
Validation	Yeast cells were stained with Alexa Fluor 488 conjugated anti-HA mAb (Cell Signaling Tech., 2350S) or Alexa Fluor 647 conjugated anti-HA mAb (Cell Signaling Tech., 3444S). Purified α1AAR in MNG/CHS was stained with Anti-FLAG-488 or -647 M1 antibody described above. Synthetic nanobody characterization was performed by flow cytometry and radioligand binding assay.

Eukaryotic cell lines

Policy information about cell lines and Sex and Gender in Research

Cell line source(s)	The Sf9 insect cell line used was obtained from Expression Systems. Yeast cells were obtained from Andrew C. Kruse lab (Harvard University). HEK293T cells were obtained from ATCC.
Authentication	No authentication required.
Mycoplasma contamination	No contamination detected.
Commonly misidentified lines (See ICLAC register)	Cells are not listed in the database.

Flow Cytometry

Plots

Confirm that:

☒ The axis labels state the marker and fluorochrome used (e.g. CD4-FITC).

☒ The axis scales are clearly visible. Include numbers along axes only for bottom left plot of group (a 'group' is an analysis of identical markers).

☒ All plots are contour plots with outliers or pseudocolor plots.

☒ A numerical value for number of cells or percentage (with statistics) is provided.

Methodology

Sample preparation	For the selection rounds 3 and 6, yeast cells were stained with Alexa Fluor-488 or -647 conjugated anti-HA antibody (Cell Signaling Tech) and 0.1 µM FLAG-tagged α1AAR with anti-FLAG M1-647 or -488. For the selection rounds 4 and 5, in order to enrich for conformational selective nanobodies, yeast cells were stained with two different populations of α1AAR labeled with anti-FLAG M1-488 and -647 fluorophores, one bound with oxymetazoline and another bound to tamsulosin.
Instrument	Accuri C6 (BD Biosciences) for analysis and FACSAria II (BD Biosciences) for sorting.
Software	FlowJo 10.
Cell population abundance	No sorting prior to analysis was done. All yeast expressing nanobody were analyzed.
Gating strategy	Standard gating was used. Yeast form a single population in an FSC/SSC plot and were gated accordingly.

☒ Tick this box to confirm that a figure exemplifying the gating strategy is provided in the Supplementary Information.
